# Computational Model of Gab1/2-Dependent VEGFR2 Pathway to Akt Activation

**DOI:** 10.1371/journal.pone.0067438

**Published:** 2013-06-21

**Authors:** Wan Hua Tan, Aleksander S. Popel, Feilim Mac Gabhann

**Affiliations:** 1 Department of Biomedical Engineering, Johns Hopkins University School of Medicine, Baltimore, Maryland, United States of America; 2 Institute for Computational Medicine and Department of Biomedical Engineering, Johns Hopkins University, Baltimore, Maryland, United States of America; University of Ulm, Germany

## Abstract

Vascular endothelial growth factor (VEGF) signal transduction is central to angiogenesis in development and in pathological conditions such as cancer, retinopathy and ischemic diseases. However, no detailed mass-action models of VEGF receptor signaling have been developed. We constructed and validated the first computational model of VEGFR2 trafficking and signaling, to study the opposing roles of Gab1 and Gab2 in regulation of Akt phosphorylation in VEGF-stimulated endothelial cells. Trafficking parameters were optimized against 5 previously published *in vitro* experiments, and the model was validated against six independent published datasets. The model showed agreement at several key nodes, involving scaffolding proteins Gab1, Gab2 and their complexes with Shp2. VEGFR2 recruitment of Gab1 is greater in magnitude, slower, and more sustained than that of Gab2. As Gab2 binds VEGFR2 complexes more transiently than Gab1, VEGFR2 complexes can recycle and continue to participate in other signaling pathways. Correspondingly, the simulation results show a log-linear relationship between a decrease in Akt phosphorylation and Gab1 knockdown while a linear relationship was observed between an increase in Akt phosphorylation and Gab2 knockdown. Global sensitivity analysis demonstrated the importance of initial-concentration ratios of antagonistic molecular species (Gab1/Gab2 and PI3K/Shp2) in determining Akt phosphorylation profiles. It also showed that kinetic parameters responsible for transient Gab2 binding affect the system at specific nodes. This model can be expanded to study multiple signaling contexts and receptor crosstalk and can form a basis for investigation of therapeutic approaches, such as tyrosine kinase inhibitors (TKIs), overexpression of key signaling proteins or knockdown experiments.

## Introduction

Vascular Endothelial Growth Factor (VEGF) signal transduction in angiogenesis is a biologically significant process both for physiological development and for pathological conditions such as cancer, ocular diseases [1–3] and ischemic diseases [4]. In particular, approved anti-angiogenic drugs have shown promise in the treatment of cancer and age-related macular degeneration. These drugs include anti-VEGF monoclonal antibodies (eg. bevacizumab, ranibizumab), tyrosine kinase inhibitors (eg. sorafenib, sunitinib) and anti-VEGF aptamers (eg. pegaptanib) [5]. Many more such drugs are in clinical trials and pre-clinical development. While the putative drug targets and primary mechanisms of action are sometimes known, secondary targets and side-effects of these drugs are difficult to predict. Furthermore, drug efficacy is highly variable, patient-specific, and largely associated with innate and acquired resistance of multiple cancer cell types [1]. VEGF acts by binding to and activating receptor tyrosine kinases (VEGFRs) on cell surfaces. The multiple signaling pathways downstream of VEGF activation of VEGFRs are dynamic and heavily coupled; cross-talk interactions between them influence cell phenotypes. Computational models have the potential to describe, explain and predict VEGF signal transduction under various conditions, including quiescence, disease and drug interventions.

The VEGF-VEGFR system is complex. In humans, it consists of five ligands (VEGF-A through -D and PlGF, placental growth factor) each with multiple isoforms, three receptor tyrosine kinases (VEGFR1, VEGFR2, VEGFR3), which actively signal as homo- and heterodimers in various combinations, and two nonsignaling co-receptors (NRP-1 and NRP-2). Here we present a mass-action ordinary differential equation (ODE) model describing VEGF-A (herein referred to as VEGF) stimulation of VEGFR2 homodimers *in vitro* because this receptor-ligand pair is primarily accountable for activating main signaling cascades in endothelial cells (eg. PI3K/Akt pathway). These pathways then lead to the activation of physiologically significant cellular processes including migration, adhesion, angiogenesis, vascular permeability, and vasodilation.

Mass action models of receptor tyrosine kinase (RTK) signaling have been developed for the ErbB or EGFR (epidermal growth factor receptor) family [6–11], and for platelet-derived growth factor receptors, PDGFRs [12–15] but less so for VEGFRs [16,17]. The above-mentioned receptors are structurally similar and their downstream effectors are similar in many ways (eg. PI3K/Akt pathway, MAPK cascade), though they often appear in different cell types. However, the biological outcomes of these signal transduction pathways may differ between cell types. Often, EGFRs in epithelial cells are associated with regulation of proliferation, PDGFRs in fibroblasts with wound healing and VEGFRs in endothelial cells with angiogenesis.

To initiate signaling, the bivalent VEGF ligand binds two VEGFR monomers. There are two possible mechanisms: ligand-induced dimerization or ligand binding to pre-dimerized receptors [18]. Upon ligation, the cytoplasmic tails of VEGFR2s autophosphorylate on residues Y1175, Y1214, and other tyrosine sites [19]. These phosphotyrosine sites then recruit adaptor molecules such as Shc and Grb2. Adaptor molecules lack enzymatic function and instead act as molecular bridges between multiple proteins. Scaffolding or docking proteins bind to adaptor proteins, joining the receptor complex. Scaffolding proteins consist of multiple binding domains and hence are generally larger than adaptor proteins [20]. Adaptor and scaffolding proteins generally contain Src homology-2 (SH2) domains that are responsible for their recruitment to the receptor at the phosphotyrosine sites [21]. These then lead to the activation of highly conserved signaling pathways such as MAPK cascade, PI3K/Akt pathway and PLCγ/PKC pathway. These pathways activate a diverse set of cellular responses e.g. proliferation, migration, apoptosis.

Differences in signal transduction between the various receptor-ligand systems can be attributed to scaffolding proteins proximal to the receptors, which are system-specific. This may then lead to differential activation of downstream pathways. *In vitro* experimental evidence suggests that Grb2-associated binder (Gab) proteins have different downstream effects in different receptor-ligand systems. In VEGF-stimulated human microvascular endothelial cells (HMVECs), siRNA experiments show that Gab1 positively regulates Akt activation, while Gab2 has the opposite effect [22,23]. In addition, Gab2 only transiently binds the receptor while Gab1 association with the receptor occurs over longer time scales [22]. In contrast, in the EGF-EGFR system, Gab1 and Gab2 positively regulate Akt activation, though interestingly the more transient binding of Gab2 relative to Gab1 is again observed [24]. 14-3-3 proteins have been shown to be responsible for mediating Gab2 dissociation from the receptor complex [25]. In the Heregulin (HRG)-Erb2/3 system, it is suggested that Gab2 negatively regulates Akt phosphorylation through negative feedback, where the dissociation of Gab2 from the receptor is mediated by Akt [26]. These three examples illustrate that Gab2 may act through distinctly different mechanisms, depending on the receptor-ligand pair. The transient nature of Gab2-receptor association renders it more difficult to study *in situ*. Hence, there is a motivation to model the kinetics of scaffolding proteins *in silico*.

Although Gab2-knockout mice are not embryonic lethal like Gab1-knockout mice are, Gab2-mediated signaling does have a functional role [20]. Gab2 has been found to be amplified and overexpressed in human breast cancer cell lines as well as in primary tumors, and was proposed to be involved in mammary carcinogenesis [27]. Gab2 has also been found to be amplified and overexpressed in melanoma, more so in metastatic than primary melanoma [28]. The relevance of Gab proteins in cell homeostasis and disease progression motivates the inclusion of both Gab1 and Gab2 proteins in signal transduction models.

**Figure 1:**
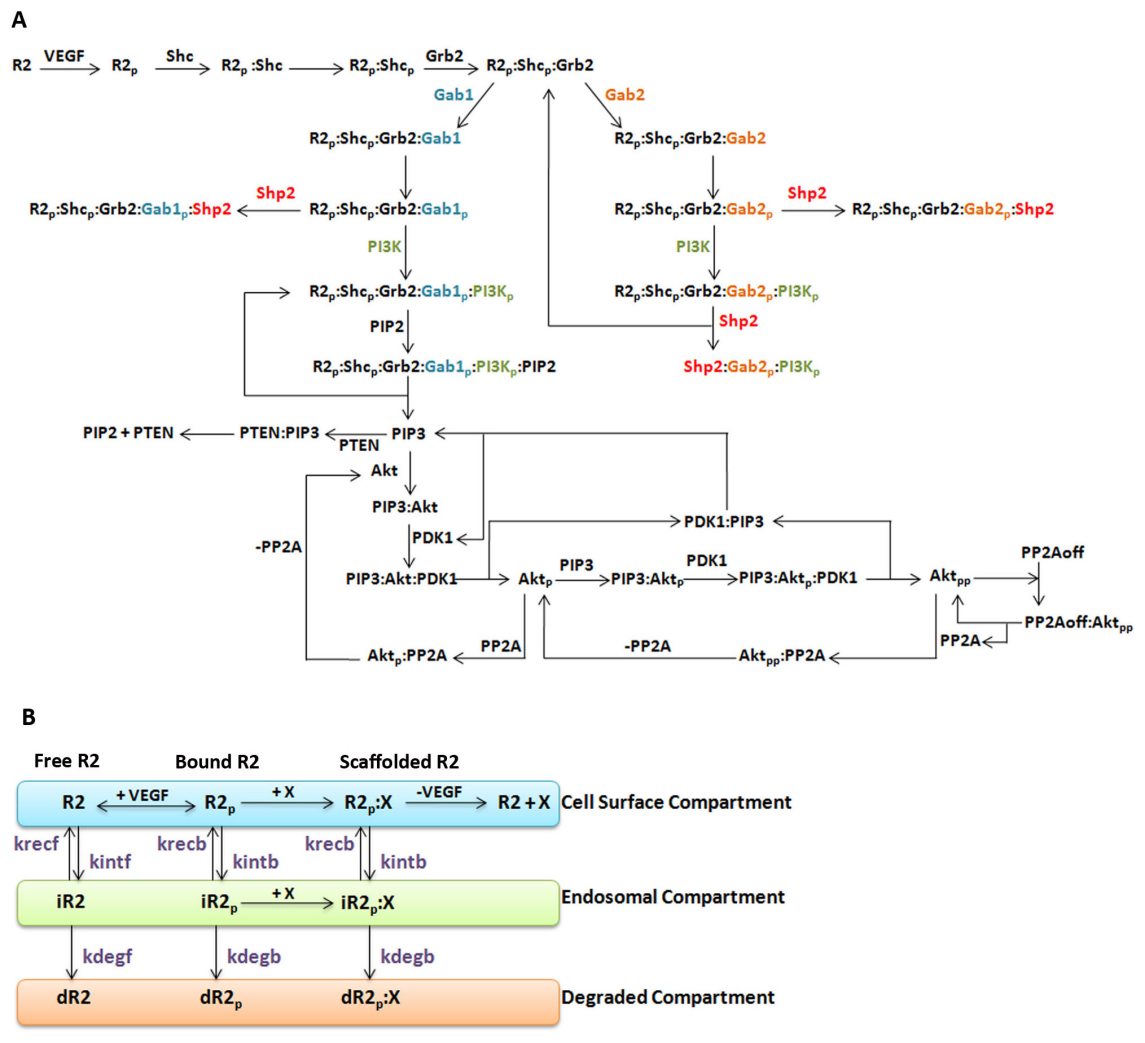
Reaction Schematic. **A**, The scaffolding proteins Gab1 and Gab2 have opposing roles in the regulation of Akt phosphorylation. Gab2 binds to the receptor complex more transiently, and its dissociation is hypothesized to be mediated by Shp2. A detailed schematic using Systems Biology Graphical Notation (SBGN) [58] [59] is shown in Figure S1 in File S1. **B**, Along with the signaling pathways, canonical pathways apply to all receptor complexes. All receptors or receptor complexes are internalized, recycled and degraded at different rates for ligated and unligated receptors. These parameters are estimated based on optimization of model outputs against experimental data. VEGF may dissociate from all VEGFR2 complexes, resulting in a disintegration of the complex. ‘iR2’ and ‘dR2’ refers to internalized and degraded receptors respectively. ‘X’ refers to any molecular species bound to VEGFR2.

The purpose of this study is to evaluate the highly transient nature of Gab2 action, which is difficult to study *in vitro*, but has significant effects on Akt phosphorylation downstream in the context of VEGF-stimulated endothelial cells [22]. The negative regulation of Akt through the dissociation of Gab2 from RTKs has been experimentally validated in the HRG-ErbB2-ErbB3 system [26] and serves as a guide to the mechanisms modeled in this study. We hypothesized that Shp2 is responsible for mediating Gab2 dissociation from the VEGFR2 receptor complex (Figure 1). This dissociation reaction results in Gab2 sequestration of PI3K, preventing further activation of Akt. As a non-receptor protein tyrosine phosphatase, Src homology-2 domain-containing phosphatase (Shp2) does not act directly to dephosphorylate VEGFR. Rather, the Shp2 binding blocks Gab1 recruitment of the p85 subunit of PI3K, and promotes signaling through the ERK pathway instead [20]. Gab2 and Shp2 interactions have been implicated in BCR/ABL transformation [29] and breast carcinogenesis [27,30].

Shp2 regulates PI3K activation differently in the Epidermal Growth Factor Receptor (EGFR), Platelet-Derived Growth Factor Receptor (PDGFR) and Insulin-like Growth Factor Receptor (IGFR) systems [31]. Such differences are a common theme in the reactions of scaffolding proteins, which have multiple binding domains and can potentially form complexes with combinations of other scaffolding proteins. Systems biology is a useful tool to understand these reactions initiated by ligand stimulation, which may be difficult to detect or analyze *in situ*. Importantly, systems biology provides an opportunity to understand which of these interactions are significant, in the context of other signaling pathways that may be activated in parallel. Such analyses may allow the identification of novel therapeutic targets. The molecular species to which the system is most sensitive may also be useful as biomarkers in guiding experimental studies and eventually in the clinical setting.

## Methods

### Formulation of biochemical reactions

To simulate *in vitro* cell culture experiments in which primary endothelial cells are stimulated with exogenously added VEGF, we developed a deterministic mass-action ODE model. In our model, aside from VEGF binding to VEGFR2, all pertinent reactions take place inside the cell, with 71 reactions involving 14 distinct proteins and 43 kinetic parameters. The interior of the cell is divided into three compartments, and molecules can move between them: the cell surface compartment; the internalized compartment (representing early endosomes); and the degraded compartment (representing irreversible entry into late endosomes/lysosomes). The list of reactions (Table S1, with initial conditions in Table S2 and kinetic parameters in Table S3, all in File S1) is based on the reaction network in Figure 1 and Figure S1 in File S1, and is compiled into a model using the MATLAB Simbiology toolbox. The system is solved using *ode15s*, one of MATLAB’s stiff ODE solvers. Here, our primary interest is in early signaling events, and the simulations produce concentration profiles of intracellular signaling molecules from 0 to 120 minutes following addition of VEGF. We assume that molecular species activated in response to VEGF stimulation have initial steady-state concentrations at zero time before the addition of VEGF. Parameter estimates are obtained from previously published mathematical models describing different receptor-ligand systems, namely VEGFR2 [32] and ErbB receptors [6–8]. The adaptor and scaffolding proteins associated with ErbB receptors are similar to those of VEGFRs as demonstrated through immunoprecipitation assays [22,23] in HUVECs.

### Molecular interactions

Biochemical reactions in this model can be broadly grouped into six modules: (1) early receptor activation events such as receptor-ligand binding and adaptor protein association; (2) reactions describing Gab1 activation of the Akt pathway; (3) proposed mechanisms for Gab2 antagonism; (4) Akt activation; (5) receptor trafficking, including internalization, endosomal sorting and degradation; and (6) VEGF dissociation from VEGFR2 complexes.

Like the PDGF system, VEGF ligands are covalently pre-dimerized. In our model, we assume that VEGFR2 are also pre-dimerized. There are multiple possible mechanisms of receptor dimerization [18], but that is not the focus in this study. Ligand stimulation is approximated as a step-activation, resulting in autophosphorylation of the VEGFR2 cytoplasmic domains. This creates docking sites for SH2-domain containing adaptor proteins Shc and Grb2. In our model, we do not distinguish explicitly between receptor autophosphorylation at the various tyrosine residues such as Y1175 and Y1214, as there is limited relevant training data.

The phosphorylation of scaffolding proteins Gab1 and Gab2 is dependent on Grb2 [20]. Based on experimental studies in VEGF-stimulated endothelial cells, the function of Gab1 is similar to that of EGF-stimulated epithelial cells [22,23,33]; Gab1 mediates PI3K activation. Hence the reactions in module 2 – the Gab1-Akt module, reactions 5-8 and 14 of Table S1 and Figure S1 in File S1-are largely adapted from existing mass action models of Gab1 activation in EGF systems [6,8]. Gab1 and Gab2 have similar binding domains, including domains for Shp2 and PI3K. Hence, we hypothesize that both Gab proteins associate with Shp2 and PI3K through similar mechanisms. However, since Gab2 binds the receptor complex more transiently than Gab1, we propose that its dissociation is mediated by Shp2, for reasons described in the introduction (Table S1 in File S1 reactions 9-13).

Akt is activated through the canonical PI3K/Akt pathway [7], where the phosphorylation of PI3K by the receptor complex catalyses the formation of PIP3 from PIP2. PIP3 production may be reversed by phosphatase PTEN. Akt phosphorylation is mediated by PDK1, whose association with PIP3 is necessary for its catalytic function. Akt is dephosphorylated by phosphatase PP2A. PP2A is activated through negative feedback by doubly phosphorylated Akt represented by Akt_pp_. These reactions are represented in Table S1 in File S1 reactions 15-30.

All receptor complexes are susceptible to receptor trafficking processes. These are primarily modeled through three kinetic parameters for each receptor complex, namely internalization, recycling and degradation, with differential rates for ligated and unligated receptors. Internalization and recycling are represented as reversible first-order reactions (Table S1 in File S1 reactions 31-44); degradation of the internalized molecules is modeled as irreversible first-order reactions (Table S1 in File S1 reactions 45-58).

Biochemical reactions in this model are written as first or second order reactions. Cross-talk interactions between the MAPK cascade and Akt cascade are not modeled here as Akt signaling has been shown to be relatively independent of the MAPK cascade [6] and hence was left out of a subsequent study on therapeutically targeting PI3K/Akt signaling in ErbB receptor systems [7].

### Model parameters

Kinetic parameters associated with signaling molecules common to RTKs (eg. Gab1, Shc, Akt etc) are taken from previously published models [6–8,32]; this reduces the number of parameters that must be estimated for these simulations (Table S3 in File S1). These signaling molecules are likely to be structurally similar regardless of ligand stimulation or resident cell-type, and the kinetics of their reactions may be conserved through different systems. On the other hand, the kinetics of reactions that involve VEGFR2 are likely to be receptor-specific, including ligand binding and receptor trafficking. VEGF-VEGFR2 binding and dissociation rates are derived from radioactive labeling experiments and Scatchard plot analysis, and have been used in previously published models [18]. VEGF receptor densities in primary endothelial cells are approximately one order of magnitude less than ErbB receptor densities in epithelial cell lines. In this model, the initial VEGFR2 density is set using quantification experiments from our group as a guide [34]. The average ligand concentration for each cell is adjusted to a cell-volume basis, and estimated using a 1 pL cell volume and 10mm cell culture medium depth. Receptor trafficking parameters, which are likely to be receptor-specific, were estimated as described in the next section.

### Parameter fitting

Receptor trafficking kinetics including receptor internalization, recycling and degradation were the only parameters for which values were not available directly from the literature or previous modeling efforts (Table S3 in File S1). These kinetic parameters were estimated by optimization of the model outputs against time-course phosphorylation profiles of VEGFR2 [35,36] and Akt [37–39] on western blots. For experimental studies where densitometric measurements are available [36–38], the time-course profile can be read directly. Where the western-blot images were published [35,39], we measured the densitometry using the ImageJ Gel Analysis tool [http://imagej.nih.gov/ij/]. Several experiments have also shown VEGFR to have different endosomal sorting characteristics from EGFR [40]. Since the experimental data provide relative and not absolute concentrations, our fitting methodology minimized least squares error between the *normalized* simulation profiles and experimental datasets (Figure S2B,D,F,H,J in File S1). The western-blot data available were semi-quantitative, and inherently relative to the control (no VEGF stimulation) densitometry. The model, in contrast, simulates absolute values of receptor densities and signaling protein concentration profiles; therefore for the purpose of comparison between the simulations and the experiments, we assumed that the maximum concentration measured in experimental data was a close approximation to the absolute maximum value in the simulated concentration profile. This is a fair assumption given that the experimental data points were collected at sufficiently short time intervals.

**Figure 2:**
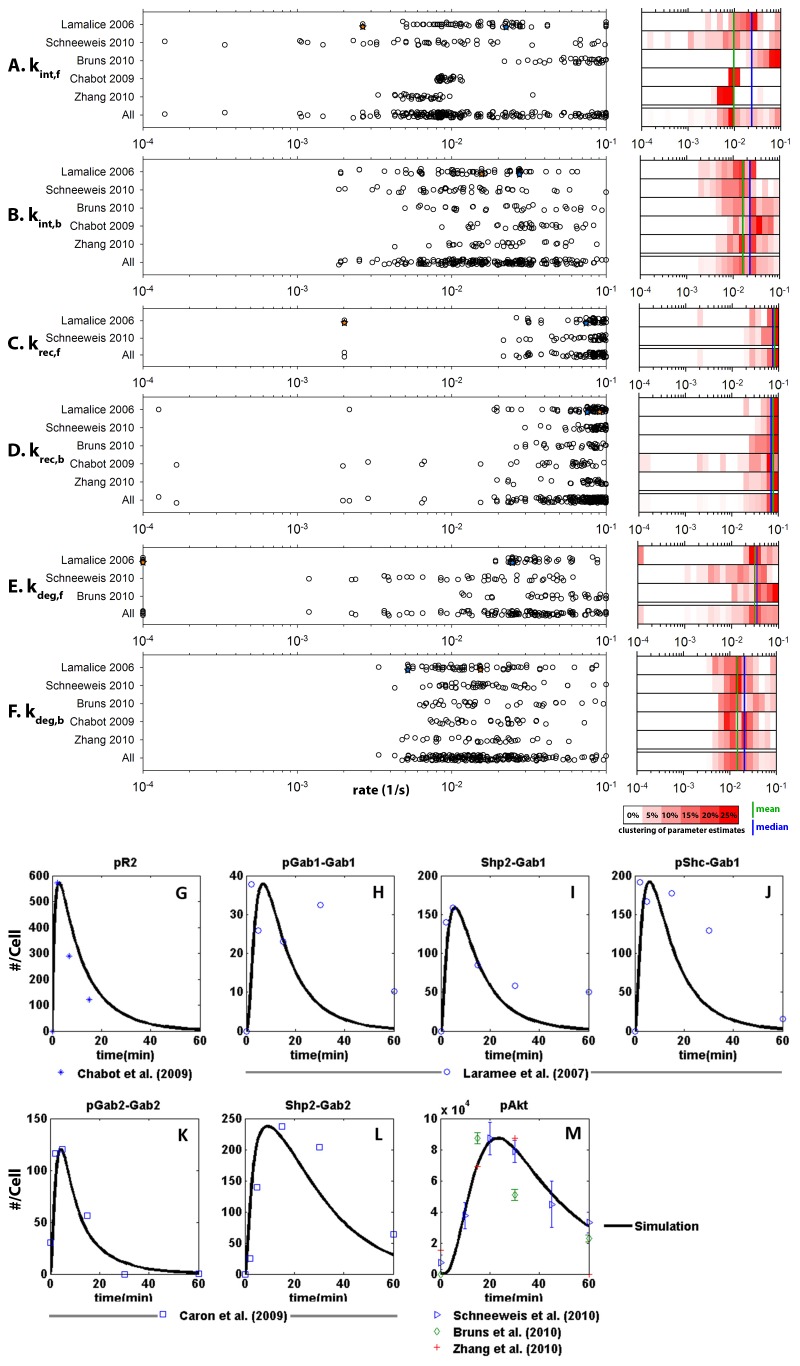
Estimation of trafficking parameters and model validation using independent experimental data. **A**–**F**, Six VEGFR2 trafficking parameters were estimated by fitting simulation results to independent time-course western blots of protein phosphorylation: Phosphorylated VEGFR2 data [35,36] and phosphorylated Akt data [37–39]. The number of fitted parameters for each dataset is limited by the number of time-points at which the metrics were measured in that dataset. For each experimental dataset, the fits were performed multiple times (Figure S2 in File S1) starting with different initial values for the parameters. The outcome is a range of estimated values for each parameter (panels A-F). We show these parameter estimates in two ways: on the left, all of the parameter estimates are shown in a dot plot, separated by experimental dataset, as well as aggregated ('All'); on the right, the estimates are binned to show the high degree of clustering of parameter estimates (plus the mean and median values). Two examples of complete parameter sets estimated by fitting to the same experimental dataset are indicated by the orange and blue stars. The distribution of estimated values for each parameter is consistent between experimental datasets. The trafficking parameters (rate constants) estimated using this method are: **A**, *kintf*, internalization of free receptors; **B**, *kintb*, internalization of bound (VEGF-ligated) receptors; **C**, *krecf*, recycling of free receptors; **D**, *krecb*, recycling of bound receptors; **E**, *kdegf*, degradation of free receptors; **F**, *kdegb*, degradation of bound receptors. **G**–**M**, Simulations recapitulate independent experimental observations of VEGFR2 and Akt phosphorylation profiles and VEGFR2 recruitment of scaffolding proteins upon VEGF stimulation. Using one of the parameter sets based on phosphorylated VEGFR2 time-course data from [36] (indicated with an orange star on panels A-F), model simulation predictions for multiple molecular species are over-laid on independent experimental data from other published sources. Western-blot time-course data were normalized to the maximum concentration computed by the model; no other fitting is done. The molecules are: **G**, All membrane-associated and internalized phosphorylated VEGFR2 complexes [35]; **H**, All complexes containing phosphorylated Gab1 (but not Shp2) [23]; **I**, All complexes in which Shp2 is bound to Gab1 [23]; **J**, All complexes in which phosphorylated Shc is bound to Gab1 [23]; **K**, All molecular complexes containing phosphorylated Gab2 [22]; **L**, All species where Shp2 is bound to Gab2 [22]; and **M**, Sum of singly and doubly phosphorylated Akt [37–39].

The optimization routine was implemented using the trust-region-reflective algorithm (*lsqnonlin* in MATLAB), and the acceptable space for the six trafficking parameters was constrained within physiologically relevant bounds (10^-1^ to 10^-4^ molecules/s). Each of the five relevant experimental datasets (phosphorylation profiles of VEGFR2 [35,36] or Akt [37–39]) was used to create at least 30 distinct sets of fitted trafficking parameters, starting from 50 runs of the simulations and discarding those giving physically unfeasible results; this resulted in 213 usable parameter sets in total. Each run used a random initial value for each parameter (within the prescribed bounds); the outcome of each run is a set of trafficking parameters that best fit that dataset. Each run produces a different set of trafficking parameters, and the resulting values for each parameter were plotted, by experimental dataset (Figure 2A–F) and with all experimental datasets aggregated ('All'). Note that since the number of parameters that can be fitted was constrained by the number of datapoints, several assumptions were made regarding the internalization, recycling and degradation rates. It was assumed that unligated and ligated receptor complexes have different internalization, recycling and degradation rates. In particular, the faster internalization rates of ligated receptors compared to unligated receptors were observed in an *in vitro* system, in the context of VEGF stimulation in bovine aortic endothelial cells (BAECs) [41]. It was further assumed that all ligated receptor complexes have the same internalization, recycling and degradation rates, regardless of internal scaffolding and complexing.

As examples of the range of parameters that were found in these best fits, two complete sets of trafficking parameters for the Lamalice et al dataset [36] are indicated with orange and blue stars (Figure 2A–F). Examining the range of values obtained for each parameter set, we note that the range of estimated values for each parameter is consistent between experimental datasets (Figure 2A–F), and that the mean and median values of these estimates is similar for each parameter (Figure 2A–F, right). These results suggest that there is a high likelihood that the true value of these parameters is within this range. To maintain consistency throughout the rest of the manuscript, and to validate these estimated values, one parameter set out of the 213 fitted was chosen to be used as a 'base case' – specifically, the set labeled with orange stars in Figure 2A–F, and listed at the end of Table S3 in File S1. The parameter set was chosen because it had the lowest sum of least squares error and the largest number of parameters fitted, based on the availability of data for 8 time-points in the dataset [36]. Note that the parameter set chosen is a single fitted set, and thus not equivalent to choosing the median for each parameter. Note also that these parameters are based only on the Lamalice et al data set [36] and therefore the other four datasets used in Figure 2A–F are independent of this and are used for validation, along with two additional datasets that provide measurements of signaling molecules intermediate between VEGFR2 and Akt. This 'base case' set of parameters is used to simulate VEGF stimulation experiments in vitro and compared to published experimental results (Figure 2G–M).

### Model Outputs

The model outputs are the concentrations of the various signaling complexes (Figure S1 in File S1) over time. To match these with experimental data, it becomes important to aggregate some complexes together. For example, due to the scaffolding and adaptor proteins, multiple molecular species in the model contain phosphorylated VEGFR2. The sum of all these is denoted ‘pR2’ (e.g. Figure 2G) and represents the population of actively signaling receptor dimers. This includes both membrane-associated receptors and internalized receptors. As noted earlier, in this model, the internalized receptors represent receptors in early endosomes while degraded receptors represent those sorted into late endosomes and marked for degradation; we assume that only the receptors at the surface and in early endsomes are signaling. A second example of aggregation is denoted ‘pAkt’ (e.g. Figure 2M), which is the sum of singly and doubly phosphorylated Akt (Akt_p_ + Akt_pp_) as both species are detected in an immunoblot.

### Sensitivity Analysis

To perform a sensitivity analysis of the model, we used the eFAST method as described by Saltelli et al [42,43] and as implemented by Kirschner and colleagues [44]. We used Simlab 2.2 from Econometrics and Applied Statistics Unit (EAS) at the Joint Research Centre (JRC) of the European Commission (Ispra, VA, Italy; http://simlab.jrc.ec.europa.eu/). The eFAST analysis method is described in more detail in the Supplemental Methods in File S1.

## Results

### Model Parameterization and Validation

To understand the VEGF-induced impact of Gab1 and Gab2 on Akt phosphorylation, we developed a mass-action model (described in *Methods*) to capture the signaling dynamics of key molecular species in the network. Due to the VEGFR-specific nature of the VEGFR trafficking parameters [40,45], and to the high sensitivity of the model outputs to these parameters (verified by analysis, see below), the trafficking parameters (Table S1 in File S1 reactions 31-58 Table S3 in File S1, parameters k_int, k_rec and k_deg) were optimized against 5 time-course phosphorylation profiles of VEGFR2 or Akt (Figure S2 in File S1), assayed in *in vitro* experiments of VEGF-stimulated endothelial cells [35–39]. As described in *Methods*, each experimental dataset provided multiple sets of parameter measurements. For each parameter, these estimates are consistent between each data set (Figure 2A–F), acknowledging that we constrained the values to an overall range of 3 orders of magnitude using the trust-region-reflective algorithm. This suggests that the 'true' values of those parameters are within these ranges. We selected a specific set of parameter estimates using only the experimental data from Lamalice et al of pVEGFR2 [36] – indicated by the orange stars – as the baseline parameters for the remainder of this study. To test the validity of this selected parameter set, we employed the four remaining datasets used in Figure 2A–F (Chabot et al for phosphorylated VEGFR2 [35]; Schneeweis et al, Bruns et al and Zhang et al for phosphorylated Akt [37–39]), plus two additional time-course experimental datasets of intermediate signaling molecules (Laramee et al for Gab1 measurements and Caron et al for Gab2 measurements [22,23]). In this way, we validate the parameterized model by comparing the outputs (concentrations of various signaling molecules, Figure 2G–M) to a total of six independent published time-course datasets. While we can estimate parameter sets from these other papers (as shown in Figure 2A–F), we used them here as validation only, validating parameters estimated using the Lamalice et al pVEGFR2 data. The model reproduced the behavior of several key nodes in the network from VEGFR2 to Akt, involving scaffolding proteins Gab1, Gab2 and their respective complexes with Shp2 (Figure 2G–M). As state variables in this model are expressed as the difference from steady state, the concentration profiles of key proteins demonstrate transient signaling, where there is an increase in signal to a maximum, followed by a return to basal levels of concentration. The attenuation of phosphorylated receptor profiles at longer time points is largely due to receptor internalization. Note, as described in the methods, the aggregation of multiple molecular species containing phosphorylated VEGFR2, denoted 'pR2' (Figure 2G), and the aggregation of singly and doubly phosphorylated Akt (Akt_p_ + Akt_pp_), denoted 'pAkt' (Figure 2M). This aggregation allows us to compare the model output with experimental results, in which the multiple phosphorylated VEGFR forms are detected together and the multiple phosphorylated Akt forms are detected together.

This model includes VEGF dissociation from VEGFR (Table S1 in File S1 Reactions 59-70), a mechanism that is not always included in RTK signaling models. Here, we assume the disintegration of the entire VEGFR-complex upon the dissociation of VEGF, which is an over-estimation for the effect of VEGF dissociation. Yet, there is little change in the key nodes of this network (Figure S3 in File S1) for typical VEGF dissociation rates (k_dV_ ~ 10^-3^/s) compared to no dissociation (k_dV_ = 0). While there is little change in the model outputs, the inclusion of dissociation-disintegration reactions shifts the sensitivity of many of the signaling complexes from being primarily sensitive to internalization to a balance of internalization and recycling rates (Figure S4 in File S1). This suggests that the sensitivity to receptor recycling is underestimated in models of RTK signaling that neglect the dynamics of ligand dissociation.

**Figure 3:**
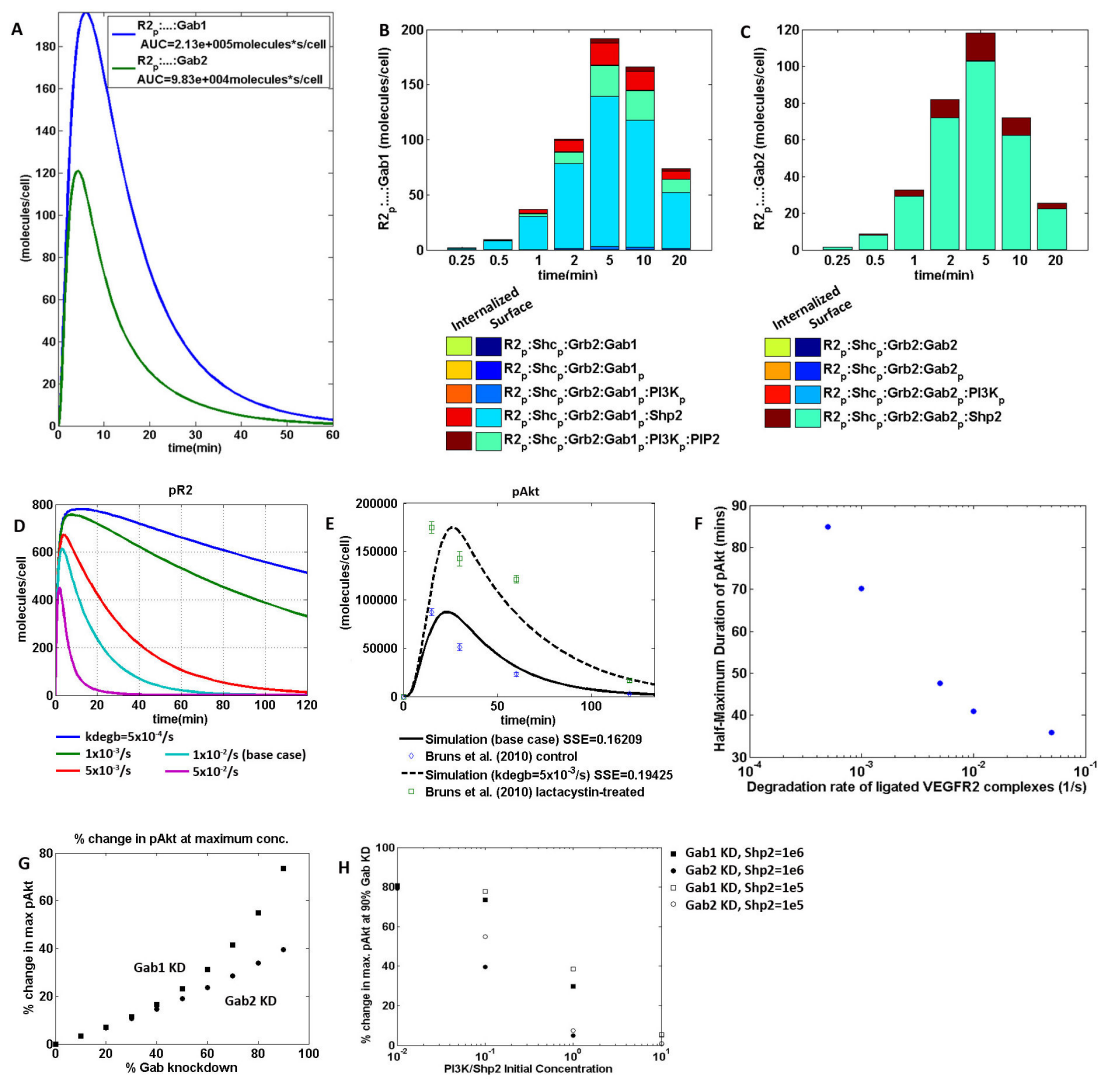
Simulations recapitulate differential Gab1/2 activation, *in vitro* experiments of lactacystin inhibiting proteasome-mediated proteolysis and knockdown of Gab1/2 using siRNA. **A**–**C**, VEGFR2 recruitment of Gab1 is greater, slower and more sustained than that of Gab2. **B** and **C**, Molecular species that make up concentration profiles in (A), at early time points. ‘Internalized’ refers to the receptor complexes in the endosomal pool and ‘Surface’ refers to plasma-membrane-associated receptor complexes on the cell surface. Model base case values are available in supplement File S1 (Tables S2 and S3 in File S1). **D**, Decreasing VEGFR2-complex degradation rates result in pR2 profiles that are larger in magnitude and more sustained. **E**, Experimental time course data of pAkt (i.e., Akt_p_ + Akt_pp_) from [37] over-laid with model simulations of decreasing VEGFR2-complex degradation rates. SSE: Sum of Squared Error, kdegb: degradation rate of VEGF-bound VEGFR2-complexes. **F**, Half-maximum duration of pAkt decreases with increasing degradation rates. **G**, Change in Akt phosphorylation in response to Gab knockdown ("KD") where PI3K/Shp2 is 0.1 and Shp2 initial concentration is at 10^6^ molecules/cell. **H**, PI3K/Shp2 ratio tunes effect of Gab knockdown on Akt phosphorylation.

We hypothesized that Shp2 associates quickly with R2_p_:…:Gab2_p_ complexes, i.e. complexes containing phosphorylated VEGFR2, phosphorylated Gab2, and other elements in between (Reaction 13, k_2dShp2) and mediates Gab2 dissociation from the VEGFR2 complex. This results in the formation of the cytosolic complex Shp2: Gab2_p_:PI3K (Table S1 in File S1), which accounts for sustained signal in the co-immunoprecipitation of Shp2-Gab2, as demonstrated experimentally [22] (Figure 2L). The kinetic rate of Shp2 association with the VEGFR2 complex in this reaction (Reaction 13, k_2dShp2) was set to be ten times that of the typical Shp2 association with Gab proteins (Reactions 8 and 12). In this case, the total concentration of Gab2-bound receptors (R2_p_:…:Gab2) reach a maximum of smaller magnitude and more quickly than Gab1-bound receptors (R2_p_:…:Gab1) (Figure 3A). The area under the curve (AUC) for R2_p_:…:Gab2 is approximately 46% of that of R2_p_:…:Gab1. This suggests a greater and more sustained response for Gab1 and a corresponding increase in Akt phosphorylation downstream. For other reactions that Gab1 and Gab2 have in common, such as with PI3K and Shp2, the kinetic parameters for Gab1 and Gab2 are the same. These concentration profiles are the sum of internalized Gab protein-bound receptor complexes as well as their membrane-bound counterparts (Figure 3B and 3C), as would be detected in an experiment co-immunoprecipitating VEGFR2 and Gab proteins. Note that a higher percentage of measured Gab1 complexes would be active signaling complexes than Gab2 complexes.

In VEGF-induced Gab protein activation, Shp2 recruitment is predicted to dominate over recruitment of the p85 subunit of PI3K in terms of magnitude (Figure S5 in File S1). In particular, Shp2 recruitment by Gab1 peaks before PI3K (Figures S5A and S5B in File S1). As is true for R2_p_:…:Gab1, R2_p_:…:Gab2 recruitment of Shp2 also dominates over the recruitment of PI3K. A similar observation was made in experiments of Gab2 recruitment of p85 and Shp2 in EGF-induced MCF-10A cells [30], where p85 recruitment was undetectable at an early stage (2.5 min after ligand stimulation).

### Inhibition of VEGFR2 degradation sustains Akt phosphorylation

VEGFR2 degradation can be attributed in part to proteasome-mediated proteolysis. To further validate model outputs, we simulate the treatment of HUVECs with lactacystin, which decreases the degradation rate of VEGFR2 (Figure 3D). As demonstrated experimentally as well as in the simulations, this results in a more sustained Akt phosphorylation (Figure 3F). In particular, these simulations suggest that a 67% decrease in VEGFR2 degradation rates relative to the control cells yields a pAkt profile (Figure 3E) closest to the experimentally measured profile [37].

### PI3K/Shp2 and Gab1/2 initial-concentration ratios influence pAkt

PI3K and Shp2 are two main binding partners of the Gab proteins. Relative concentrations between kinases and phosphatases in a cell can be significant in influencing system behavior. In this proposed mechanism, Gab1 and Gab2 compete for PI3K. Intuitively, the concentration of PI3K in the endothelial cell is important in influencing the topology of this network. The ratio of initial concentrations of PI3K and Shp2 needs to be less than one (i.e. PI3K < Shp2) for Gab2 knockdown to have an appreciable negative effect on Akt phosphorylation (Figure 3H). This was consistent over two orders of magnitude of initial enzyme concentrations, suggesting that within the range of physiologically relevant concentrations of PI3K and Shp2, the ratio of kinase to phosphatase is more important in influencing system behavior than the absolute concentrations. At higher concentration ratios of PI3K/Shp2 in the cell, the lipid kinase is not limiting and Akt is phosphorylated to the same extent regardless of Gab2 knockdown. This implies that sufficient PI3K is activated for maximal Akt phosphorylation.

To study the effect of siRNA knockdown experiments *in vitro*, we simulated a range of decreases in Gab protein initial concentrations. As demonstrated in [22], an experimental knockdown of Gab1 decreased pAkt by 32% at 10 minutes and 24% at 20 minutes. Here, our model predicts that for a 90% KD of Gab1, maximum pAkt decreases by 73.5%. Similarly, in the experimental knockdown of Gab2, pAkt increases 46% at 10 min and 66% at 20 mins relative to the corresponding time points in the control experiment. Here, the model predicts a maximum 39.6% increase in pAkt compared to the control. These figures serve to demonstrate that the model recapitulates semi-quantitative experimental data within reasonable variability. We also studied the input-output characteristics between Gab protein concentration and Akt phosphorylation. Following Gab1 knockdown in our model, a log-linear relationship was predicted between a decrease in Akt phosphorylation and decrease in Gab1 concentration, but a linear relationship between a Gab2 knockdown and an increase in Akt phosphorylation (Figure 3G).

**Figure 4:**
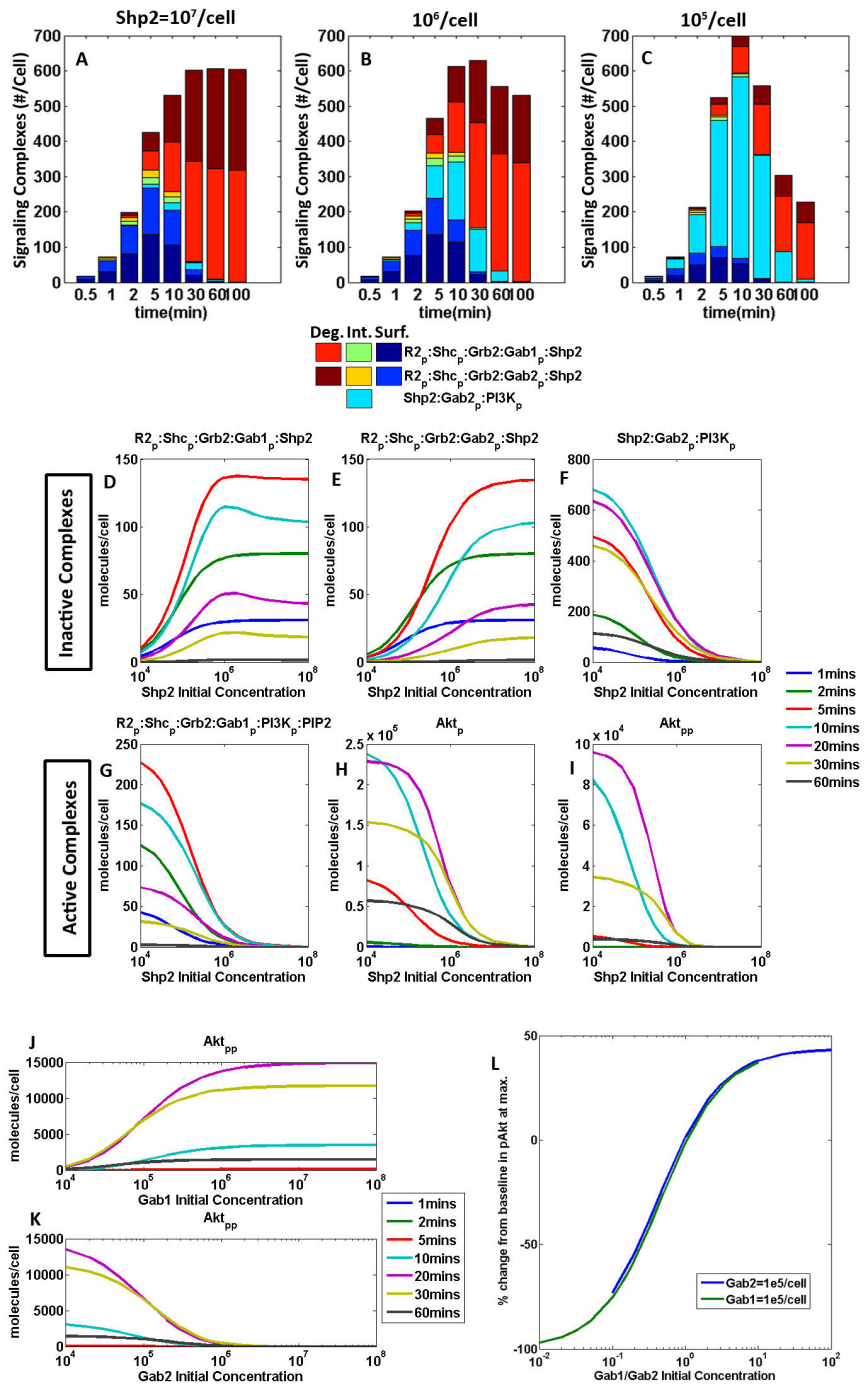
Akt phosphorylation is sensitive to the initial concentrations of Shp2 and Gab1/2. **A**–**I**, Decreasing the initial concentration of Shp2 increases the total proportion of free Shp2 consumed. Different biochemical reactions dominate with changes in initial Shp2 concentration. **A**, Shp2 Initial Concentration at 10^7^ molecules/cell; **B**, at 10^6^ molecules/cell; **C**, at 10^5^ molecules/cell. ‘Deg’ refers to receptor complexes sorted into late endosomes and eventually degraded. ‘Int.’ refers to internal, includes the receptor complexes in the endosomal pool and cytosol. ‘Surf.’ refers to plasma-membrane-associated receptor complexes on the cell surface. **D**–**I**, Sensitivity to initial Shp2 concentration of Akt-activating species (D–F) and Akt-nonactivating species (G–I). Positive slopes indicate an increase in concentration of that molecule with increasing Shp2. Concentrations are in units of molecules/cell. **J**–**L**, The ratio of concentrations of Gab1 to Gab2 influences Akt phosphorylation. **J** and **K**, The dependence of the concentration of doubly phosphorylated Akt on the initial concentrations of (J) Gab1 and (K) Gab2 initial concentrations in various time points. **L**, Predicted maximum Akt phosphorylation, relative to the baseline simulation ([Gab1] = [Gab2] = 10^5^/cell). Akt phosphorylation increases with increasing value of the ratio of Gab1/Gab2 initial concentration. The ratio appears to be more important than the individual values of Gab1 and Gab2, as shown by changing the ratio by varying Gab1 concentration (blue line, constant Gab2) and by varying Gab2 concentration (green line, constant Gab1).

The initial concentration of Shp2 is also important in the proposed mechanism. Decreasing the initial concentration of Shp2 increases the total proportion of free Shp2 consumed in the various biochemical reactions (Figure 4A–C). This seems counter-intuitive, since the presence of more reactants should yield more products. However, in this network, there are 3 reactions that involve free Shp2 (Reactions 8, 12, 13 of Table S1 in File S1). The dominance of each reaction is dependent on the initial concentration of Shp2. In a high-Shp2 regime, the formation of R2_p_: Shc_p_: Grb2: Gab2_p_: Shp2 is proportionally large (Figure 4A, brown, yellow and blue). On the other hand, in a low-Shp2 regime, the formation of Shp2: Gab2_p_:PI3K_p_ is proportionally large (Figure 4C, cyan). This indicates that in the low Shp2 regime, the pathway through R2_p_: Shc_p_: Grb2: Gab2_p_:PI3K_p_ dominates, and leads to the feedback reaction, which recycles R2_p_: Shc_p_: Grb2 and forms Shp2: Gab2_p_:PI3K_p_ (Figure 1). This recycling effect results in the presence of more active receptor complexes, which can go down multiple possible pathways and one possibility is the production of more Shp2-containing complexes. Although reactions involving Shp2 and Gab2 generally repress Akt activation, the model predicts that the transient binding of Gab2 and hence the recycling of R2_p_: Shc_p_: Grb2 can, depending on the mass fluxes at particular instants in time through the simulation, partially promote Akt activation.

In general, the dependence of molecular complexes on Shp2 initial concentration generally reflects whether or not that particular molecular complex promotes active Akt phosphorylation. Molecular complexes that are Akt-activating have a negative slope (Figure 4G–I), in other words, they decrease with increasing Shp2 concentration. In contrast, Akt-deactivating species have a positive slope (Figure 4D-E), increasing with increasing Shp2 concentration, with the exception of Shp2: Gab2_p_:PI3K_p_. While Shp2: Gab2_p_:PI3K_p_ sequesters PI3K in the cytosol, hence reducing Akt activation, the plot for Shp2: Gab2_p_:PI3K_p_ shows a negative slope (Figure 4F), which is typical of an Akt-activating signaling molecule. The recycling of R2_p_: Shc_p_: Grb2 is coupled with the formation of Shp2: Gab2_p_:PI3K_p_ and this reaction is partly Akt-activating.

Given that Gab1 and Gab2 have opposite roles upon VEGF stimulation in HMVECs, it is intuitive to ask: what is the threshold ratio of Gab1: Gab2 protein initial concentrations that would render one completely dominant over the other? Local sensitivity of Gab1 or Gab2 initial concentration with respect to Akt_pp_ suggests that a ratio of ten-fold would render one Gab protein substantially dominant over the other (Figure 4J–L). The basal level of each Gab protein is 10^5^/cell in this model. Interestingly, given different kinetics between Gab1 and Gab2 association with the receptor complex, their initial concentration ratio influences Akt phosphorylation through several orders of magnitude of Gab protein concentration, and the ratio of concentrations is more determinative than the absolute concentration of either molecule (Figure 4L).

### System is more sensitive to VEGF concentration than VEGFR2 density at a single cell level

**Figure 5:**
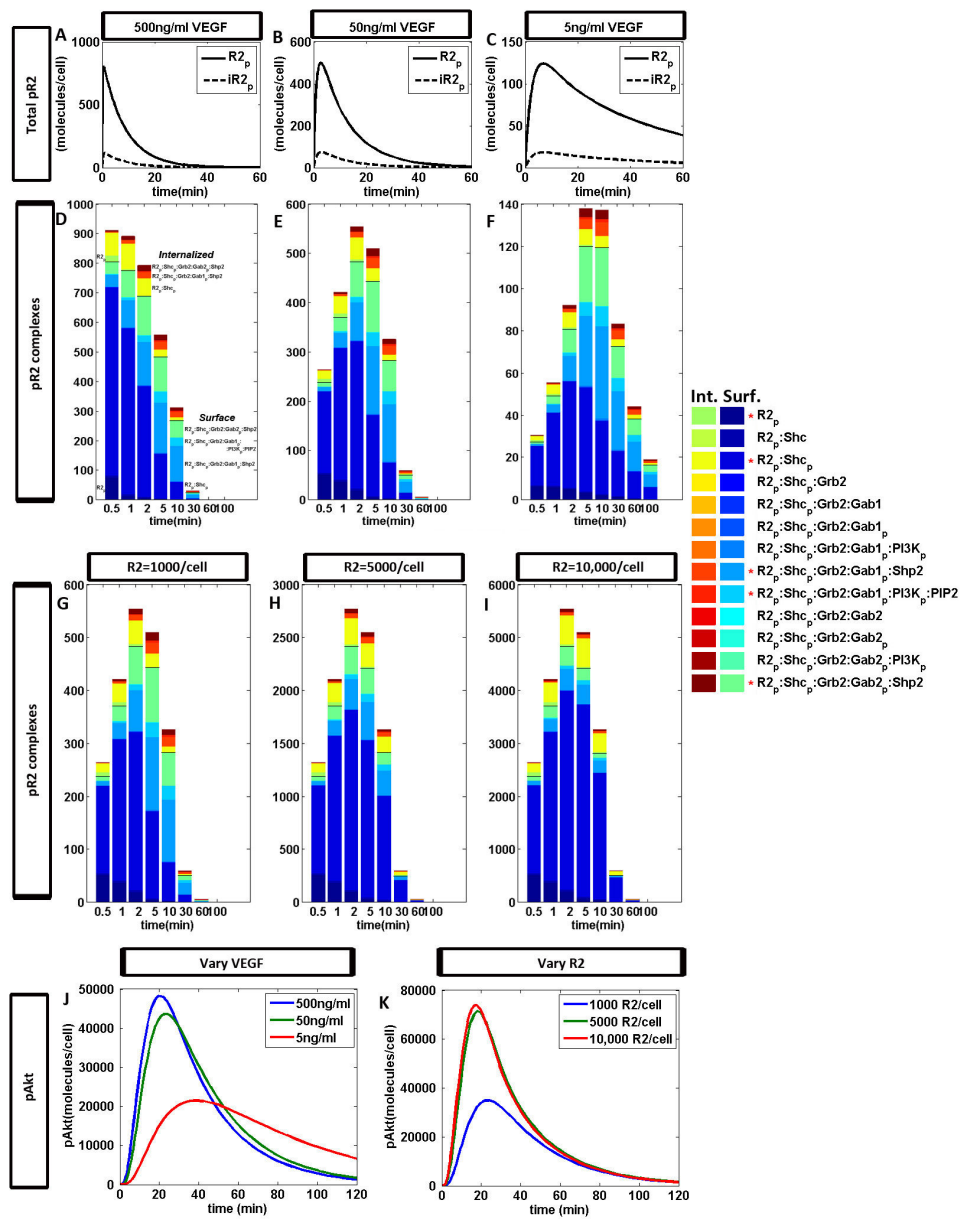
Effect of receptor-ligand kinetics on Akt phosphorylation. **A**–**F**, Receptor phosphorylation dynamics in response to VEGF concentrations: (A,D) 500 ng/ml; (B,E) 50 ng/ml; and (C,F) 5ng/ml. R2_p_ refers to plasma membrane-associated receptors, iR2_p_ refers to internalized receptors. Estimates of VEGF concentration based on a 1 picoliter cell volume. **G**–**I**, Receptor phosphorylation dynamics in response to different VEGFR2 densities: **G**, 1000 VEGFR2-dimers/cell; **H**, 5,000 VEGFR2-dimers/cell; and **I**, 10,000 VEGFR2-dimers/cell. **J** and **K**, Sum of singly and doubly phosphorylated Akt corresponding to simulations in panels A-F (J) and panels G-I (K). R2 here refers to dimerized VEGFR2 receptors. ‘Int.’ refers to the receptor complexes in the endosomal pool and ‘Surf.’ refers to plasma-membrane-associated receptor complexes on the cell surface.

Changes to the exogenous VEGF concentration affects the magnitude and time-to-peak of receptor phosphorylation profiles (Figure 5A–F), while altered VEGFR2 density only affects its magnitude (Figure 5G–I; note the different y-axis scales). Specifically, decreasing VEGF concentration decreases the magnitude of the receptor phosphorylation profile and delays its time-to-peak. Furthermore, decreasing VEGF concentration increases the proportion of larger receptor complexes internalized, such as R2_p_: Shc_p_: Grb2: Gab1_p_:PI3K_p_:PIP2 (Figure 5D–F). This occurs because ligated receptors are internalized and recycled faster than unligated receptors. Hence, with lower ligand concentrations, net receptor internalization rates are lower, and larger membrane-associated receptor complexes can form before internalization occurs. VEGF ligand availability is dynamic due to receptor trafficking. This may account for the variability in time-to-peak in VEGFR2 phosphorylation profiles as measured in published cell culture experiments, despite similar VEGF concentrations being used.

Recent studies in VEGFR trafficking have demonstrated that it is not simply a mechanism for signal downregulation, which had been the conventional assumption. Rather, it facilitates *different* signaling mechanisms that have significant impacts on cell physiology [46,47]. Hence the distribution (cell surface/intracellular) of receptors in the endothelial cell may be an important metric, indicative of the extent of activation of the various downstream pathways. In our simulations, the ratio of the concentrations of cell-surface receptors and internalized receptors were consistent at all ligand concentrations. Over 100 minutes after addition of VEGF, the time-integrated concentration of cell-surface receptors was calculated to be ~86% of the total receptor population. Experimental studies also show that internalized pool of VEGFR2 is not negligible, both in quiescent [45] and stimulated [40] states. Since receptor trafficking is dynamic and ligand-dependent, variability is expected.

Decreasing receptor density is predicted to impact only the magnitude of receptor phosphorylation profiles but not the timing (Figure 5G–I, note the different y-axis scales); the phosphorylated VEGFR2 profile scales linearly with initial VEGFR2 density. The proportion of the various receptor complexes also remains largely constant regardless of receptor density. This may be because model presented here is a single-receptor-type, single-cell model; at the tissue level [48], in the presence of multiple receptor types and co-receptors (neuropilins), the sensitivity of receptor densities will differ.

Interestingly, Akt phosphorylation profiles remain largely robust to order-of-magnitude changes in receptor density and ligand concentration (Figure 5J-K). In *in vitro* experiments, 50-100ng/ml are the highest concentrations of VEGF used; 5-20 ng/ml are typical. Here, the simulations indicate that there are saturation thresholds of ligand concentration and receptor density for maximal Akt activation.

### Modular Sensitivity Analysis

**Figure 6:**
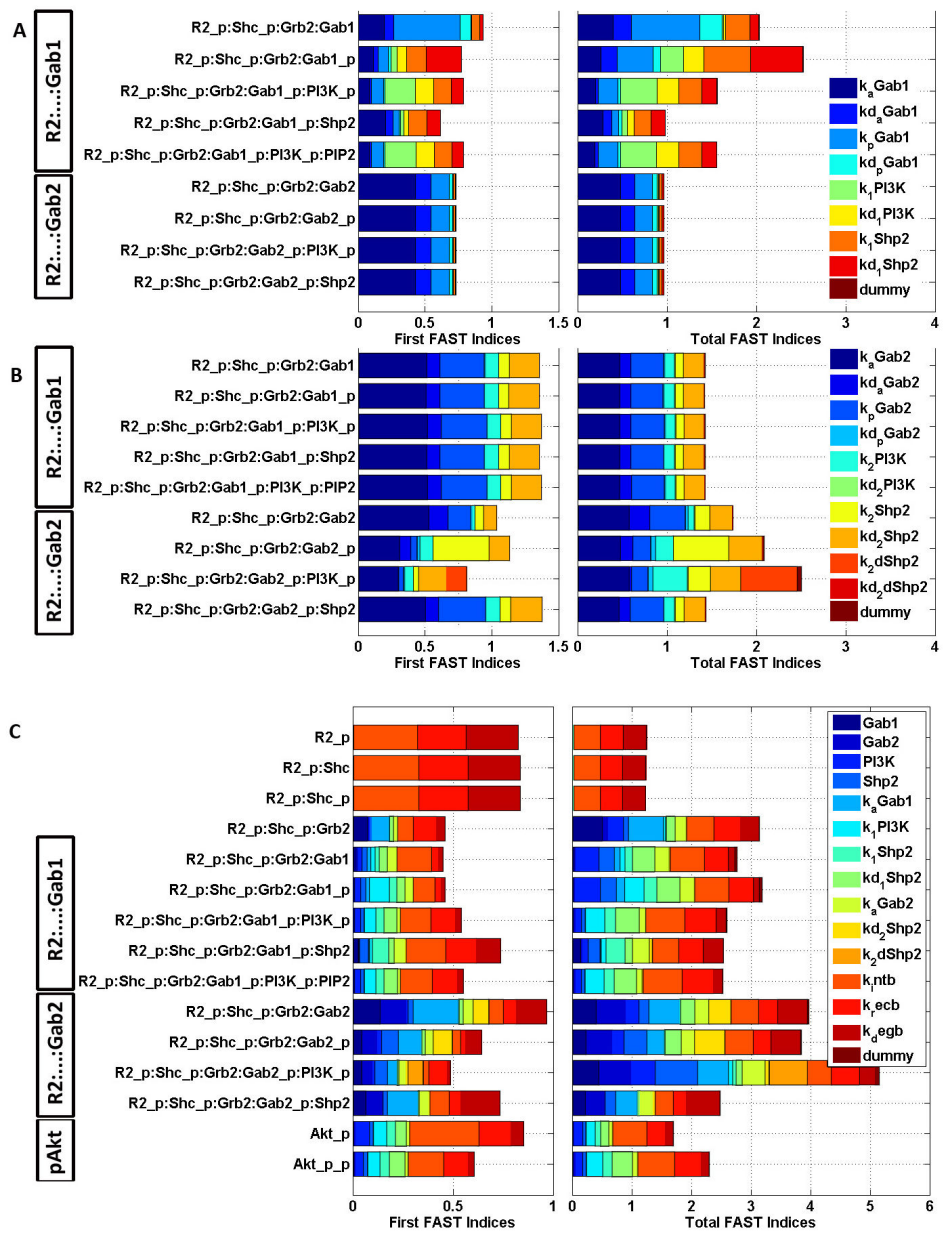
eFAST First and Total sensitivity Indices for select parameters and molecular species. Internalized counterparts of receptor complexes not presented here have identical sensitivity indices as their membrane-associated counterparts. **A**, R2_p_ :…:Gab1 and R2_p_:…:Gab2 sensitivity to kinetic parameters in the Gab1 module. **B**, R2_p_ :…:Gab1 and R2_p_:…:Gab2 sensitivity to kinetic parameters in the Gab2 module. **C**, eFAST analysis on the initial-concentration and kinetic parameters shown to be most sensitive on each of their respective modular eFAST analyses.

Parameters in this system were organized into several modules and Extended Fourier Amplitude Sensitivity Test (eFAST) analysis [42,43] was performed to quantify interactions between parameters in each module. Phosphorylation-dephosphorylation signal transduction systems such as these are often characterized as insulators, due to the fast time-scales of these reactions relative to the decay of the inputs [49]. Insulated modules act relatively independently of upstream inputs and downstream outputs. eFAST analysis was performed for the some of the most sensitive parameters from each module to quantify interactions between modules, if any. The Total FAST Index of an input is the normalized sum of its variance and covariances with other inputs, in all possible combinations, with respect to an output (see Supplemental Methods in File S1). As such, it is not possible to show which specific higher order interactions dominate for each input parameter. Given this non-identifiable nature of the eFAST Total Indices and the complexity of this model, limiting the number of parameters in each eFAST analysis avoids the computation of spurious interactions between parameters in the system. Specifically, the sets of parameters investigated were as follows: initial concentrations of unique proteins in the system (Figure S6 in File S1); kinetic parameters in reactions involving Gab1 (Figure 6A); kinetic parameters in reactions involving Gab2 (Figure 6B); VEGFR2 trafficking kinetics (Supplemental Figure S4 in File S1); and finally, a combination of more sensitive parameters from the aforementioned analyses (Figure 6C). Sensitivity indices were computed for all the receptor complexes (plasma membrane-associated and internalized) and for Akt (singly and doubly phosphorylated). Sensitivity indices of internalized receptor complexes are not presented here because they were identical to their membrane-associated counterparts, as expected.

A 'dummy' parameter, i.e. one that does not appear in the model, is introduced in the eFAST analysis to quantify the extent of aliasing in the analysis [44] (Figure 6). Dummy parameters can have non-negligible FAST indices when none of the input parameters are important to a particular output; for example, kinetic parameters in the Gab1 and Gab2 modules (e, g, k_a_Gab1, k_a_Gab2) have little effect on the formation of small VEGR2 complexes such as R2_p_: Shc and R2_p_: Shc_p_ (Figure 6C). When the FAST indices are normalized, the parameters (including the dummy) tend to have large but fairly equal FAST indices throughout, indicating a small signal to noise ratio.

The sensitivity of signaling molecules to initial concentration parameters is important, because protein concentrations often depend on cell types and culture conditions. Using the eFAST analysis, receptor density appears to be the most significant of all initial-concentration parameters (Figure S6 in File S1). Based on the stoichiometry in the model, VEGFR2 is limiting and changes to receptor density scales the receptor phosphorylation profile linearly (Figure 5G–I). VEGF concentration affects the duration of the signal to a greater extent, which is not captured in this analysis based on time-integrated data. Receptor density and initial ligand concentrations have a large impact on small receptor complexes (R2_p_: Shc, R2_p_: Shc_p_), but VEGFR2 complexes bound to more than one adaptor molecule (Shc) are predicted to be almost completely insulated from the changes in VEGF concentration across 2 orders of magnitude. This verifies the simulations that demonstrate a robust Akt phosphorylation response, regardless of changes in the receptor phosphorylation profile (Figure 5). Interestingly, the R2_p_:…:Gab1 complex is more sensitive to initial concentrations of Shp2 and PI3K than to Gab1; but initial concentrations of both Gab1 and Gab2 are important (along with PI3K and Shp2) in influencing R2_p_:…:Gab2. In fact, Gab1 initial concentrations appears to influence the formation of R2_p_:…:Gab2, more than it does R2_p_:…:Gab1. These results indicate asymmetry between the Gab1 and Gab2 modules, although many of their binding partners, reactions and reaction rates are similar.

Similarly, the Gab1 and Gab2 modules are differently sensitive to their kinetic parameters (Figure 6A-B), another indication that the system is not symmetric. For example, of the Gab-module kinetic parameters, R2_p_:…:Gab2 is most sensitive to k_a_Gab2 (Figure 6B, dark blue), while R2_p_:…:Gab1 is least sensitive to k_a_Gab1 (Figure 6A, dark blue). Conversely, R2_p_:…:Gab1 is most sensitive to k_1_PI3K (Figure 6A, green) but R2_p_:…:Gab2 is least sensitive to k_2_PI3K (Figure 6B, cyan), with the exception of R2_p_: Shc_p_: Grb2: Gab2_p_:PI3K_p_. Parameters in reactions involving Gab1 generally have a smaller and non-synergistic effect on molecular species involving Gab2 (Figure 6A, bottom four rows). The same is observed for parameters in reactions involving Gab2 and Gab1-parameters. The quick phosphorylation rates of Gab1 and Gab2 (k_p_Gab1, k_p_Gab2) may contribute to the insulation between Gab1 and Gab2 modules.

The ranking of Gab2-module parameters to which each R2_p_:…:Gab2 complex is sensitive is different (Figure 6B). In comparison, more similarities in ranking are observed in the Gab1 module. Interestingly, receptor complex formation is least sensitive to the parameters governing the dissociation of Gab2 from the receptor complex (k_2_dShp2 and kd _2_dShp2) (Figure 6B), suggesting that the proposed dissociation reaction mediated by Shp2 (Reaction 13) recapitulates experimental data but has a small effect on the overall system behavior. This is encouraging, since these parameters have no precedent estimates in the literature.

The kinetic parameter for the dissociation of Gab2 from the receptor complex (k_2_dShp2) is one to which all molecular species except R2_p_: Shc_p_: Grb2: Gab2_p_:PI3K appear to be insensitive; both the first-order and total eFAST indices for k_2_dShp2 and R2_p_: Shc_p_: Grb2: Gab2_p_:PI3K are high, and the difference between the Total and First indices for this input-output pair is largest amongst all other pairs in this analysis. This means that k_2_dShp2 interacts with other parameters in Gab2-module reactions to exert an impact on R2_p_: Shc_p_: Grb2: Gab2_p_:PI3K (Figure 6B).

Finally, to understand which parameters the signaling complexes are most sensitive to, we conducted an eFAST analysis on a selection of those parameters to which the signaling complexes were shown to be most sensitive in each of their respective modular eFAST analyses. The selected parameters include initial concentrations, kinetic parameters in the Gab1 and Gab2 modules, as well as the VEGFR2 trafficking module. The signaling complexes are most sensitive to the trafficking parameters involving the internalization, recycling and degradation of pR2 (Figure 6C). This result demonstrates the necessity in fitting the trafficking parameters against experimental data.

**Figure 7:**
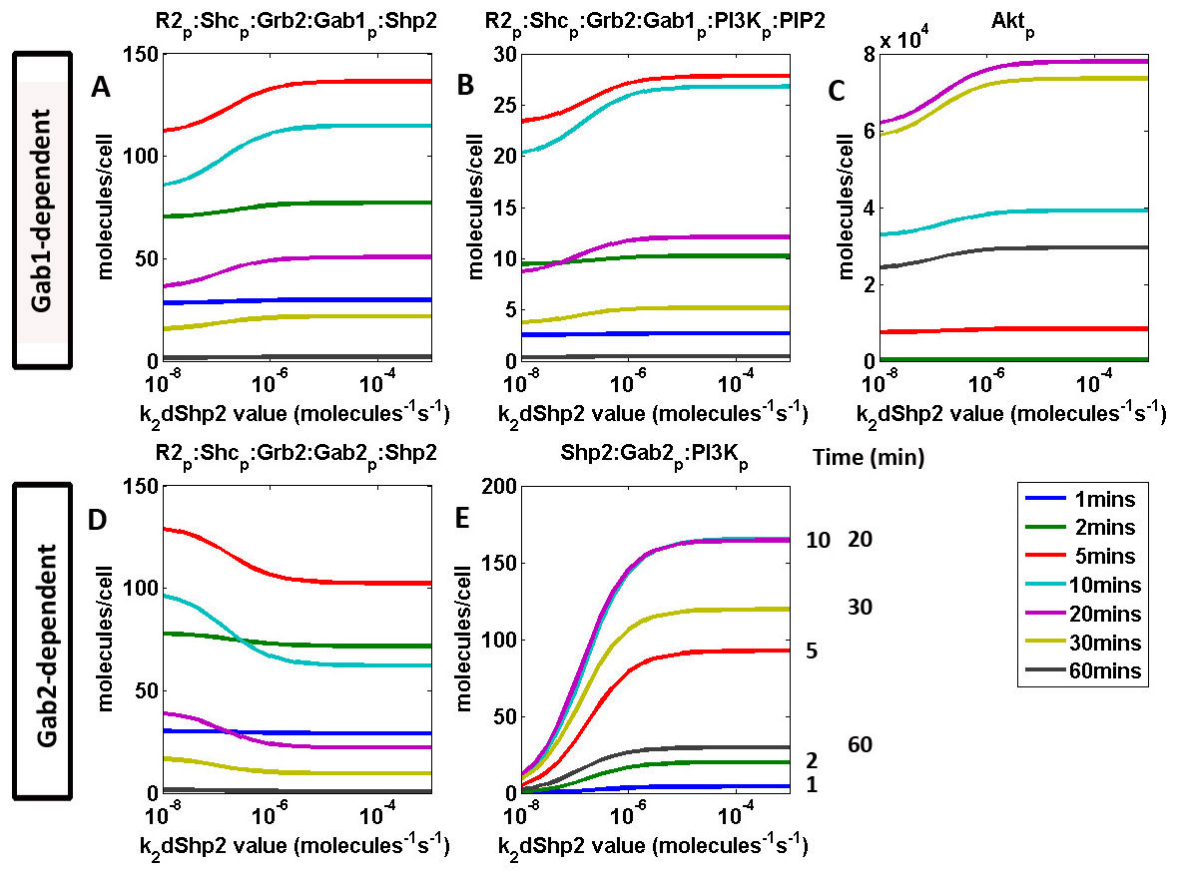
Sensitivity of model outputs to Gab2 dissociation rate from VEGFR2 complex. **A**–**B**, Sensitivity of Gab1-associated VEGFR2 complexes, **C**, Sensitivity of singly phosphorylated Akt, **D-E**, Molecular species in the Gab2 module. **D**, Sensitivity of R2_p_: Shc_p_: Grb2: Gab2_p_: Shp2, an inactive Gab2-associated VEGFR2 complex and **E**, Sensitivity of cytosolic complex Shp2: Gab2_p_ :PI3K_p_.

Model outputs show that the rate of dissociation of Gab2 from the receptor complex (k_2_dShp2) affects the distribution between Gab1- and Gab2-associated VEGFR receptors (Figure 7). However, above a threshold of about 10^-4^/molecules/s, a faster dissociation rate ceases to have an effect on the system. Figure 7 demonstrates that this holds through time and that the parameter does not result in a marked delay in time-to-peak. Molecular complexes that form as a result of Gab1 association with the receptor (Figure 7A–C), regardless of whether it is signal-downregulating (Figure 7A) or signal-activating (Figure 7B), increase with increasing dissociation rate (k_2_dShp2). It follows that with increased formation of R2_p_: Shc_p_: Grb2: Gab1_p_:PI3K_p_:PIP2 at faster k_2_dShp2 rates, Akt phosphorylation follows the same trend, being downstream of the former. Increasing dissociation rates of Gab2 from the receptor (kd _a_Gab2) decreases the proportion of Gab2-bound receptors (Figure 7D), while increasing the formation of the inactive cytosolic complex Shp2: Gab2_p_ : PI3K_p_, byproduct of the dissociation reaction.

## Discussion

We described here the first deterministic mass-action model that describes and predicts part of the VEGFR2 signaling network in a comprehensive fashion. It is also the first to describe both Gab1 and Gab2 in modulating downstream signaling. One of the challenges of developing models of signal transduction is defining its scope: the level of detail necessary to recapitulate experimental evidence, yet tractable in complexity. The use of fundamental first- and second-order kinetic equations here rather than Michaelis-Menten or Hill approximations allow the study of signaling input-output characteristics of modules in this network. Importantly, it provides more flexibility in the demarcation of these modules, and facilitates the understanding of specific reactions in the context of others. This model focuses on the opposite roles of scaffolding proteins Gab1 and Gab2. While ubiquitous in intracellular systems, scaffolding proteins exhibit different behavior in regulation of major signaling pathways, specific to the receptor-ligand pair. A more mechanistic understanding of the VEGFR2 signaling pathways in the context of these scaffolding proteins is crucial in devising both anti- and pro-angiogenic therapies for cancer and ischemia respectively.

In this study, we show that differences in kinetics of Gab1 and Gab2 scaffolding proteins affect Akt phosphorylation to different extents. VEGFR2 recruitment of Gab1 is greater in magnitude, slower, and more sustained than that of Gab2. As Gab2 transiently binds VEGFR2 complexes, there is a recycling of VEGFR2 complexes, which can then continue to participate in other signaling pathways, possibly Akt-activating or -deactivating. Correspondingly, a log-linear relationship was observed between a decrease in Akt phosphorylation and Gab1 knockdown while a linear relationship was observed between an increase in Akt phosphorylation and Gab2 knockdown. Another theme in this study is the significance of initial-concentration ratios of antagonistic molecular species Gab1/Gab2 (Figure 4L) in determining Akt phosphorylation profiles regardless of each protein’s concentration, within physiologically relevant ranges. However, when two antagonistic pairs, Gab1/Gab2 and PI3K/Shp2, were studied simultaneously (Figure 3H), their effect on pAkt profiles was less predictable.

The objective for developing such a computational model is not to predict concentration profiles or estimate parameters with good accuracy but rather, to predict, in a semi-quantitative fashion, a system-wide response to cellular interventions of biological interest. These may include the therapeutic administration of molecular drugs such as tyrosine kinase inhibitors (TKIs), overexpression of key signaling proteins or knockdown experiments. Although this model was trained with semi-quantitative data from five published experimental datasets in different *in vitro* set-ups, we have observed order-of-magnitude-consistent optimization estimates within the physiologically relevant range for these parameters, spanning three orders of magnitude. This suggests that the parameters can reasonably be expected to reside within this narrower range of values (Figure 2A–F). Furthermore, this model recapitulates experimental data describing profiles of scaffolding protein interactions in response to VEGF stimulation [22,23], as well as interventions involving inhibition of proteasome-mediated proteolysis of VEGFR2 and Gab1/Gab2 siRNA experiments [22]. It has been shown that poorly constrained parameters are inherent to large nonlinear ODE models unless highly accurate and complete datasets are available [50]. Such datasets are difficult to obtain with the current state-of-the-art technology. Nonetheless, this model may be used to identify parameters to which the key signaling molecular complexes are sensitive, warranting further experimental investigation. The topology of this model is also congruous with several experimental observations both in *in vitro* VEGF systems as well as other RTK systems [7,11,15,51,52].

In this model, eFAST was used as the primary tool for sensitivity analysis, which allows for the assessment of parameter sensitivity over a larger and statistically defined parameter space. Total FAST indices compare the sum of variances of each input and covariances between each input and all combinations of other inputs, with variance of each output. Hence, parameters with much larger total FAST indices compared with first FAST indices indicate that the parameter is important in concert with other parameters but less so on its own. Notably, one such parameter (k_2_dShp2) in this model accounted for the dissociation of Gab2 from the VEGFR2 complex, suggesting that the parameter should be studied in tandem with the rest of the parameters in the Gab2 module. In addition, we have shown that internalization and degradation kinetics of VEGFR2 are particularly sensitive and impact downstream Akt phosphorylation.

This model describes a competition between Gab1 and Gab2, for PI3K and hence the control of Akt activation. Arguably, competition between Gab1 and Gab2 can also occur at the level of PIP3 and Akt. The pleckstrin homology (PH) domain is well conserved between Gab family proteins and binds PIPs. PIPs account for membrane recruitment of Gab proteins. Lacking an intrinsic catalytic unit, PIP3 is a less active node in the pathway than PI3K. Competition between Gab1 and Gab2 for Akt is less likely as there is limited evidence of Akt-Gab protein binding. Akt-Gab2 association has been reported once [26] and Akt-Gab1 association has not been observed [20]. Another limitation in this model is the inclusion of VEGFR2 homodimers as the only VEGFR type. VEGFR1 is less well understood than VEGFR2; it has a lower kinase activity than VEGFR2, but does have a role in regulating VEGF-stimulated signaling [39].

Validation of computational models relies on experimental data; detailed quantitative experimental methods are relevant to the future development of this and related models. For example, the ubiquity of phosphorylation in signaling dynamics may require more high-throughput experimental studies for an unbiased understanding. Recently, mass spectrometry has been used to study time-dependent phosphoproteomics in the EGFR [53], HER2 [54] and IGFR signaling networks [55]. While conventional western blots are reliable and relatively accurate, their measurement is hypothesis-driven, and the likelihood of finding novel signaling proteins and pathway crosstalks is smaller. Nevertheless, western blots are still invaluable in the verification of results from mass spectrometry. The use of less specific phosphotyrosine antibodies is necessary for such global experimental studies. As a result, there is lower accuracy and the need for additional validation. The identification of new biomarkers strongly correlated with cellular processes such as migration and proliferation [54] can be useful in the construction of models predictive of such processes important to angiogenesis. In addition, this technology can be extended to study patient-specific drug response from a phosphoproteomic perspective [55].

Given the setup of this model, it is possible to extend it to describe in greater detail VEGFR trafficking mechanisms and their signaling consequences, as well as other canonical pathways such as MAPK cascade and PLCϒ/PKC and their crosstalk mechanisms. With recent experimental evidence, trafficking mechanisms have been shown to be distinct from other RTKs [40,45,56] and significant in endothelial cell physiology [46,47]. The paradigm of receptor trafficking as an indispensable signaling mechanism adds a new layer of complexity that renders these systems less predictable. For example, Protein Tyrosine Phosphatase-1B (PTP-1B), localized on the endoplasmic reticulum, has anti- and pro-signaling roles in RTK signaling; anti-signaling in the dephosphorylation of RTKs, and pro-signaling in the downregulation of multivesicular body (MVB) formation in late endosomal sorting [57]. Systems biology is necessary in describing this dynamic interplay between trafficking and signaling mechanisms, pro- and anti-signaling components, kinases and phosphatases, altogether.

This model is the first to simulate VEGF receptor-specific intracellular signaling. The model can recapitulate certain key aspects of VEGF receptor signaling that are distinct from other receptor tyrosine kinases, notably the behavior of Gab1 and Gab2 in influencing Akt activation. This is important, because targeting VEGF pathways in disease requires a specific understanding, rather than therapies generically targeting proteins that are downstream of many receptors. In addition, a key observation of the models is that certain concentration ratios are more important than individual concentrations in the behavior of the intracellular signaling system. This has implications for personalized medicine, for example, in which biomarkers may constitute more than one protein or gene. The model developed here also includes VEGFR trafficking mechanisms and therefore can be expanded to study multiple signaling contexts (e.g. cell surface vs. intracellular signal initiation) as well as receptor crosstalk. The model can also form a basis for investigation of therapeutic approaches, such as tyrosine kinase inhibitors, overexpression of key signaling proteins or knockdown therapies.

## Supporting Information

File S1 Supporting information for this study. This file contains Table S1-S3, which list the reactions and parameters used in the computational model. It also contains Figure S1-S6, which contain a detailed schematics, as well as additional results figures. Finally, it includes one section of supplemental methods, describing the sensitivity analysis for this study.
(PDF)
