# Supplementary material for: Computational Model of Gab1/2-Dependent VEGFR2 Pathway to Akt Activation
Source: PLoS One. 2013 Jun 21;8(6):e67438. doi: 10.1371/journal.pone.0067438 (PMC3689841; doi:10.1371/journal.pone.0067438)
Supplement: File S1 — This file contains Table S1-S3, which list the reactions and parameters used in the computational model. It also contains Figure S1-S6, which contain a detailed schematics, as well as additional results figures. Finally, it includes one section of supplemental methods, describing the sensitivity analysis for this study. (PDF) [file pone.0067438.s001.pdf]

Supplement to

Computational Model of Gab1/2-dependent VEGFR2 pathway to Akt activation

Wan Hua Tan[1], Aleksander S. Popel[1], Feilim Mac Gabhann[2]

[1] Department of Biomedical Engineering, School of Medicine, Johns Hopkins University, Baltimore, Maryland, 21205 USA

[2] Institute for Computational Medicine and Department of Biomedical Engineering, Johns Hopkins University, Baltimore, Maryland, 21218 USA

Contents:

Table S1. Biochemical Reactions

Table S2. Initial Concentrations (molecules/cell)

Table S3. Kinetic Parameters

Figure S1: Schematic of reactions represented in Systems Biology Graphical Notation (SBGN).

Figure S2: Trafficking parameter fits from five independent datasets.

Figure S3: Local Sensitivity analysis of VEGF dissociation rate from VEGFR2- complexes show that at physiologically relevant ranges, these reactions have a small effect on signaling.

Figure S4: eFAST analysis of VEGFR2 trafficking parameters and Akt-phosphorylation parameters show that trafficking parameters are more sensitive.

Figure S5: Shp2 dominates recruitment by Gab proteins.

Figure S6. eFAST sensitivity indices of initial- concentration parameters.

Supplemental Methods – Parameter Sensitivity

Note: Citation numbers in this document refer to the bibliography in the main document.

Table S1. Biochemical Reactions

Symbols

:	complex formation
_p	phosphorylation at a residue
i	internalized molecular species
d	degraded molecular species
<->	reversible reaction
->	irreversible reaction

	Reaction	Forward parameter	Backward parameter
**Module 1: Early Receptor Activation**			
1	R2 + V <-> R2_p	k_pR2	kd_pR2
2	R2_p + Shc <-> [R2_p:Shc]	k_aShc	kd_aShc
3	[R2_p:Shc] <-> [R2_p:Shc_p]	k_pShc	kd_pShc
4	[R2_p:Shc_p] + Grb2 <-> [R2_p:Shc_p:Grb2]	k_Grb2	kd_Grb2
**Module 2: Gab1 Module**			
5	[R2_p:Shc_p:Grb2] + Gab1 <-> [R2_p:Shc_p:Grb2:Gab1]	k_aGab	kd_aGab
6	[R2_p:Shc_p:Grb2:Gab1] <-> [R2_p:Shc_p:Grb2:Gab1_p]	k_pGab1	kd_pGab1
7	[R2_p:Shc_p:Grb2:Gab1_p] + PI3K <-> [R2_p:Shc_p:Grb2:Gab1_p:PI3K_p]	k_1PI3K	kd_1PI3K
8	[R2_p:Shc_p:Grb2:Gab1_p] + Shp2 <-> [R2_p:Shc_p:Grb2:Gab1_p:Shp2]	k_1Shp2	kd_1Shp2
14	[R2_p:Shc_p:Grb2:Gab1_p:PI3K_p] + PIP2 <-> [R2_p:Shc_p:Grb2:Gab1_p:PI3K_p:PIP2]	k_aPIP2	kd_aPIP2
**Module 3: Gab2 Module**			
9	[R2_p:Shc_p:Grb2] + Gab2 <-> [R2_p:Shc_p:Grb2:Gab2]	k_aGab	kd_aGab
10	[R2_p:Shc_p:Grb2:Gab2] <-> [R2_p:Shc_p:Grb2:Gab2_p]	k_pGab1	kd_pGab1
11	[R2_p:Shc_p:Grb2:Gab2_p] + PI3K <-> [R2_p:Shc_p:Grb2:Gab2_p:PI3K_p]	k_1PI3K	kd_1PI3K
12	[R2_p:Shc_p:Grb2:Gab2_p] + Shp2 <-> [R2_p:Shc_p:Grb2:Gab2_p:Shp2]	k_1Shp2	kd_1Shp2
13	[R2_p:Shc_p:Grb2:Gab2_p:PI3K_p] + Shp2 <-> [R2_p:Shc_p:Grb2] + [Shp2:Gab2_p:PI3K_p]	k_2dShp2	kd_2dShp2

Table S1 Continued.

	Reaction	Forward parameter	Backward parameter
**Module 4: Akt cascade**			
15	[R2_p:Shc_p:Grb2:Gab1_p:PI3K_p:PIP2] -> [R2_p:Shc_p:Grb2:Gab1_p:PI3K_p] + PIP3	k_fPIP3	
16	PIP3 + PTEN <-> [PIP3:PTEN]	k_aPTEN	kd_aPTEN
17	[PIP3:PTEN] -> PIP2 + PTEN	k_fPIP2	
18	PIP3 + Akt <-> [PIP3:Akt]	k_aAkt	kd_aAkt
19	[PIP3:Akt] + PDK1 <-> [PIP3:Akt:PDK1]	k_aPDK1	kd_aPDK1
20	[PIP3:Akt:PDK1] -> Akt_p + [PIP3:PDK1]	k_fAkt_p	
21	[PIP3:PDK1] -> PIP3 + PDK1	k_fPIP3PDK1	
22	PIP3 + Akt_p <-> [PIP3:Akt_p]	k_aAkt	kd_aAkt
23	[PIP3:Akt_p] + PDK1 <-> [PIP3:Akt_p:PDK1]	k_aPDK1	kd_aPDK1
24	[PIP3:Akt_p:PDK1] -> Akt_p_p + [PIP3:PDK1]	k_fAkt_p	
25	Akt_p_p + PP2A <-> [Akt_p_p:PP2A]	k_aPP2A	kd_aPP2A
26	[Akt_p_p:PP2A] -> Akt_p + PP2A	k_fAkt_pPP2A	
27	Akt_p + PP2A <-> [Akt_p:PP2A]	k_aPP2A	kd_aPP2A
28	[Akt_p:PP2A] -> Akt + PP2A	k_fAkt_pPP2A	
29	Akt_p_p + PP2Aoff <-> [Akt_p_p:PP2Aoff]	k_aPP2Aoff	kd_aPP2Aoff
30	[Akt_p_p:PP2Aoff] -> Akt_p_p + PP2A	k_fPP2A	
**Module 5a: Trafficking (Internalization and Recycling)**			
31	R2 <-> iR2	k_intf	k_recf
32	R2_p <-> iR2_p	k_intb	k_recb
33	[R2_p:Shc] <-> [iR2_p:Shc]	k_intb	k_recb
34	[R2_p:Shc_p] <-> [iR2_p:Shc_p]	k_intb	k_recb
35	[R2_p:Shc_p:Grb2] <-> [iR2_p:Shc_p:Grb2]	k_intb	k_recb
36	[R2_p:Shc_p:Grb2:Gab1] <-> [iR2_p:Shc_p:Grb2:Gab1]	k_intb	k_recb
37	[R2_p:Shc_p:Grb2:Gab1_p] <-> [iR2_p:Shc_p:Grb2:Gab1_p]	k_intb	k_recb

Table S1 Continued.

Reaction		Forward parameter	Backward parameter
38	[R2_p:Shc_p:Grb2:Gab1_p:PI3K_p] <-> [iR2_p:Shc_p:Grb2:Gab1_p:PI3K_p]	k_intb	k_recb
39	[R2_p:Shc_p:Grb2:Gab1_p:Shp2] <-> [iR2_p:Shc_p:Grb2:Gab1_p:Shp2]	k_intb	k_recb
40	[R2_p:Shc_p:Grb2:Gab2] <-> [iR2_p:Shc_p:Grb2:Gab2]	k_intb	k_recb
41	[R2_p:Shc_p:Grb2:Gab2_p] <-> [iR2_p:Shc_p:Grb2:Gab2_p]	k_intb	k_recb
42	[R2_p:Shc_p:Grb2:Gab2_p:PI3K_p] <-> [iR2_p:Shc_p:Grb2:Gab2_p:PI3K_p]	k_intb	k_recb
43	[R2_p:Shc_p:Grb2:Gab2_p:Shp2] <-> [iR2_p:Shc_p:Grb2:Gab2_p:Shp2]	k_intb	k_recb
44	[R2_p:Shc_p:Grb2:Gab1_p:PI3K_p:PIP2] <-> [iR2_p:Shc_p:Grb2:Gab1_p:PI3K_p:PIP2]	k_intb	k_recb
**Module 5b: Trafficking (Degradation)**			
45	iR2 -> dR2	k_degf	
46	iR2_p -> dR2_p	k_degb	
47	[iR2_p:Shc] -> [dR2_p:Shc]	k_degb	
48	[iR2_p:Shc_p] -> [dR2_p:Shc_p]	k_degb	
49	[iR2_p:Shc_p:Grb2] -> [dR2_p:Shc_p:Grb2]	k_degb	
50	[iR2_p:Shc_p:Grb2:Gab1] -> [dR2_p:Shc_p:Grb2:Gab1]	k_degb	
51	[iR2_p:Shc_p:Grb2:Gab1_p] -> [dR2_p:Shc_p:Grb2:Gab1_p]	k_degb	
52	[iR2_p:Shc_p:Grb2:Gab1_p:PI3K_p] -> [dR2_p:Shc_p:Grb2:Gab1_p:PI3K_p]	k_degb	
53	[iR2_p:Shc_p:Grb2:Gab1_p:Shp2] -> [dR2_p:Shc_p:Grb2:Gab1_p:Shp2]	k_degb	
54	[iR2_p:Shc_p:Grb2:Gab2] -> [dR2_p:Shc_p:Grb2:Gab2]	k_degb	
55	[iR2_p:Shc_p:Grb2:Gab2_p] -> [dR2_p:Shc_p:Grb2:Gab2_p]	k_degb	
56	[iR2_p:Shc_p:Grb2:Gab2_p:PI3K_p] -> [dR2_p:Shc_p:Grb2:Gab2_p:PI3K_p]	k_degb	
57	[iR2_p:Shc_p:Grb2:Gab2_p:Shp2] -> [dR2_p:Shc_p:Grb2:Gab2_p:Shp2]	k_degb	
58	[iR2_p:Shc_p:Grb2:Gab1_p:PI3K_p:PIP2] -> [dR2_p:Shc_p:Grb2:Gab1_p:PI3K_p:PIP2]	k_degb	

Table S1 Continued.

	Reaction	Forward parameter	Backward parameter
**Module 6: VEGF dissociation**			
59	[R2_p:Shc] -> V + R2 + Shc	kd_v	
60	[R2_p:Shc_p] -> V + R2 + Shc	kd_v	
61	[R2_p:Shc_p:Grb2] -> V + R2 + Shc + Grb2	kd_v	
62	[R2_p:Shc_p:Grb2:Gab1] -> V + R2 + Shc + Grb2 + Gab1	kd_v	
63	[R2_p:Shc_p:Grb2:Gab1_p] -> V + R2 + Shc + Grb2 + Gab1	kd_v	
64	[R2_p:Shc_p:Grb2:Gab1_p:PI3K_p] -> V + R2 + Shc + Grb2 + Gab1 + PI3K	kd_v	
65	[R2_p:Shc_p:Grb2:Gab1_p:Shp2] -> V + R2 + Shc + Grb2 + Gab1 + Shp2	kd_v	
66	[R2_p:Shc_p:Grb2:Gab2] -> V + R2 + Shc + Grb2 + Gab2	kd_v	
67	[R2_p:Shc_p:Grb2:Gab2_p] -> V + R2 + Shc + Grb2 + Gab2	kd_v	
68	[R2_p:Shc_p:Grb2:Gab2_p:PI3K_p] -> V + R2 + Shc + Grb2 + Gab2 + PI3K	kd_v	
69	[R2_p:Shc_p:Grb2:Gab2_p:Shp2] -> V + R2 + Shc + Grb2 + Gab2 + Shp2	kd_v	
70	[R2_p:Shc_p:Grb2:Gab1_p:PI3K_p:PIP2] -> V + R2 + Shc + Grb2 + Gab1 + PI3K + PIP2	kd_v	
71	[Shp2:Gab2_p:PI3K_p] -> Shp2 + Gab2 + PI3K	kd_v	

Table S2. Initial Concentrations (molecules/cell)

Protein	Concentration (molecules/cell)	Source
R2	1.00E+03	Within range measured in [34]
V	4.29E+06	Estimated based on experimental setup [22,23]
Shc	1.10E+06	[6]
Grb2	1.27E+03	[6]
Gab1	1.00E+05	[6]
PI3K	1.00E+05	[8]
Shp2	1.00E+06	[6]
Gab2	1.00E+05	Estimate
PIP2	7.00E+05	[7]
PTEN	3.50E+05	[7]
Akt	9.00E+05	[7]
PDK1	9.50E+05	[7]
PP2A	4.00E+03	[7]
PP2Aoff	6.40E+04	[7]

Table S3. Kinetic Parameters.

These parameters can each apply to multiple reactions, as denoted in Table S1.

Kinetic rate	Value	Units	Source
k_pR2	2.58E-09	cell/molecules/s	[32]
kd_pR2	1.00E-03	/s	[32]
k_aShc	1.39E-07	cell/molecules/s	[6]
kd_aShc	1.00E-01	/s	[6]
k_pShc	6.00E+00	/s	[6]
kd_pShc	6.00E-02	/s	[6]
k_Grb2	6.73E-06	cell/molecules/s	[6]
kd_Grb2	1.66E-04	/s	[6]
k_aGab	6.67E-05	cell/molecules/s	[6]
kd_aGab	1.00E+00	/s	[58]
k_pGab1	1.87E+00	/s	[58]
kd_pGab1	1.00E+00	/s	[6]
k_1PI3K	1.50E-05	cell/molecules/s	[6]
kd_1PI3K	2.00E-01	/s	[6]
k_1Shp2	3.33E-05	cell/molecules/s	[6]
kd_1Shp2	1.00E-01	/s	[6]
k_2dShp2	3.33E-04	/s	estimation
kd_2dShp2	1.00E-06	/s	estimation
k_aPIP2	5.00E-06	cell/molecules/s	[7]
kd_aPIP2	1.00E-01	/s	[7]
k_fPIP3	2.00E-01	/s	[7]
k_aPTEN	5.00E-06	cell/molecules/s	[7]
kd_aPTEN	1.00E-01	/s	[7]
k_fPIP2	1.00E-01	/s	[7]
k_aAkt	2.60E-04	cell/molecules/s	[7]
kd_aAkt	1.00E-01	/s	[7]
k_aPDK1	6.70E-05	cell/molecules/s	[7]
kd_aPDK1	1.00E-01	/s	[7]
k_fAkt_p	1.00E+00	/s	[7]
k_fPIP3PDK1	2.00E-01	/s	[7]
k_aPP2A	1.70E-06	cell/molecules/s	[7]
kd_aPP2A	1.00E-01	/s	[7]
k_fAkt_pPP2A	1.50E+00	/s	[7]
k_aPP2Aoff	8.30E-09	cell/molecules/s	[7]
kd_aPP2Aoff	5.00E-01	/s	[7]
k_fPP2A	1.00E-01	/s	[7]

Table S3 continued.

Kinetic rate	Value	Units	Source
k_intf	2.65E-03	/s	estimation
k_recf	2.02E-03	/s	estimation
k_intb	1.56E-02	/s	estimation
k_recb	9.06E-02	/s	estimation
k_degf	1.00E-04	/s	estimation
k_degb	1.54E-02	/s	estimation
kd_v	1.00E-03	/s	[32]

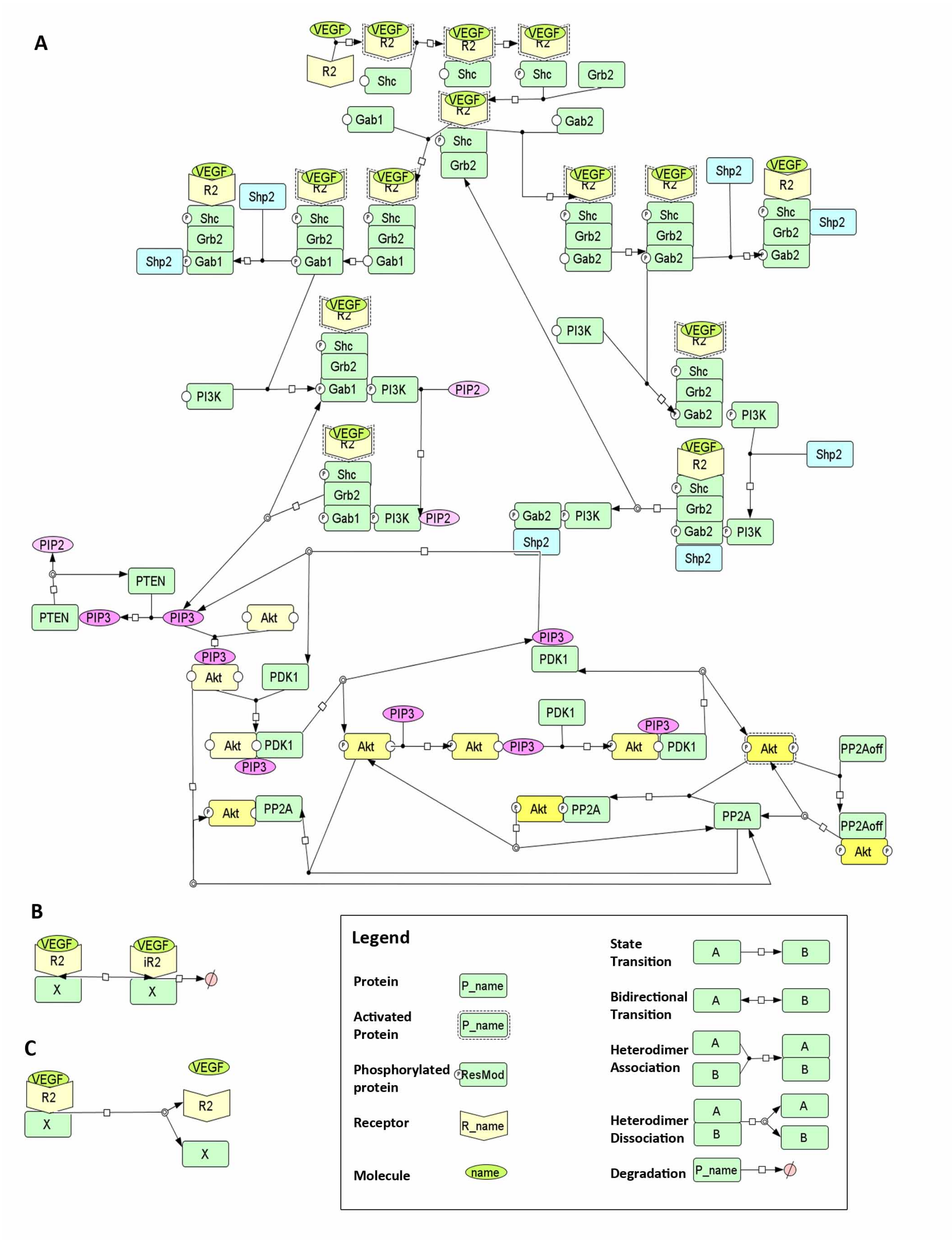
A
B
C
Legend
Protein
Activated Protein
Phosphorylated protein
Receptor
Molecule
State Transition
Bidirectional Transition
Heterodimer Association
Heterodimer Dissociation
Degradation
P_name
ResMod
R_name
name
VEGF
R2
iR2
Shc
Grb2
Gab1
Gab2
Shp2
PI3K
PIP2
PIP3
PTEN
Akt
PDK1
PP2A
PP2Aoff

Figure S1

Figure S1: Schematic of reactions represented in Systems Biology Graphical Notation (SBGN) [58]**. This figure** provides a more detailed look at the signaling network, using the commonly-accepted Systems Biology Graphical Notation (SBGN) [58] to represent the biochemical reactions. **A**, The scaffolding proteins Gab1 and Gab2 have opposing roles in the regulation of Akt phosphorylation. Gab2 binds to the receptor complex more transiently, and its dissociation is hypothesized to be mediated by Shp2. **B and C**, Along with the signaling pathways, two canonical pathways apply to all receptor complexes. **B**, All receptors or receptor complexes are internalized, recycled and degraded at different rates for ligated and unligated receptors. These parameters are estimated based on optimization of model outputs against experimental data. **C**, VEGF may dissociate from all VEGFR2 complexes, resulting in a disintegration of the complex. 'iR2' and 'dR2' refers to internalized and degraded receptors respectively. 'X' refers to any molecular species bound to VEGFR2. This figure was created using CellDesigner [59].

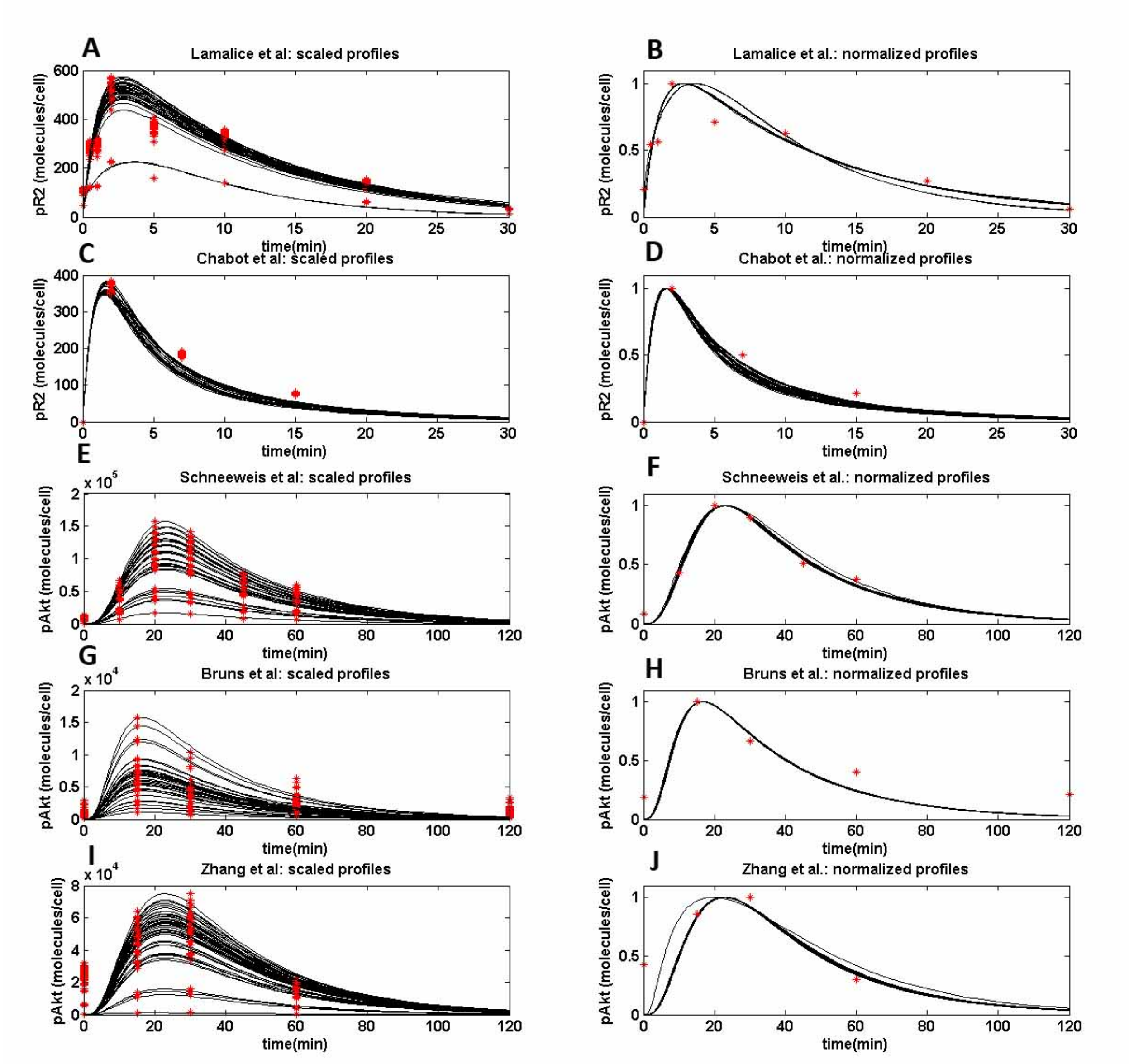

Figure S2: Trafficking parameter fits from five independent datasets. Trafficking parameters are estimated by minimizing the difference between the normalized simulation outputs and experimental time-points. The range of estimates for each trafficking parameter, as depicted in Figure 2A-F of the main manuscript, results from the ability to obtain multiple best fits, based on different sets of parameter values, to the same five experimental datasets. The right-hand panels show the experimental datapoints (red symbols) with the simulated output data for each parameter set (black lines) normalized to the maximum of each experimental dataset. The left-hand panels show a different view of the same

data, with the simulated data shown as absolute values of predicted protein phosphorylation for each parameter set, with the experimental datapoints scaled to the maximum of each profile. (A-B) 50 parameter sets fitted to phosphorylated VEGFR2 (pR2) data at 8 time-points [36]. (C-D) 40 parameter sets fitted to pR2 data at 4 time-points [37]. (E-F) 40 parameter sets fitted to phosphorylated Akt (pAkt) data at 6 time-points [39]. (G-H) 33 parameter sets fitted to pAkt data at 5 time-points [39]. (I-J) 50 parameter sets fitted to pAkt data at 4 time-points [39].

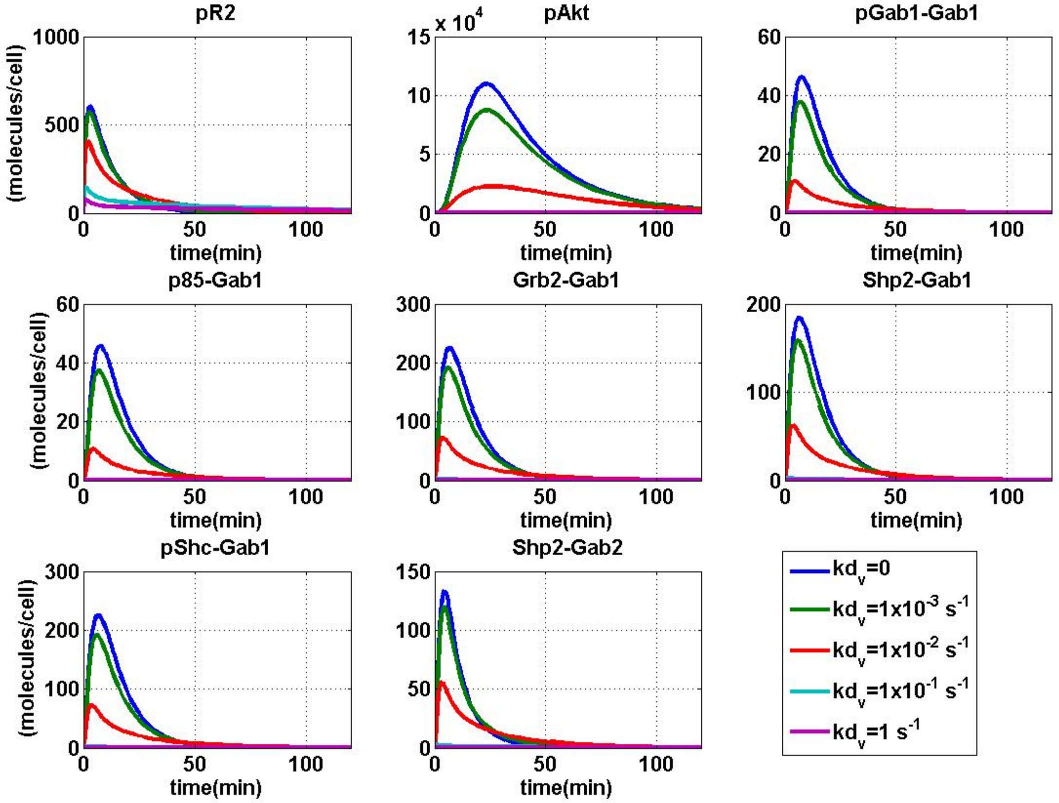

Figure S3: Local Sensitivity analysis of VEGF dissociation rate from VEGFR2- complexes show that at physiologically relevant ranges, these reactions have a small effect on signaling. For VEGF, the typical rate is 10^{-3} /s.

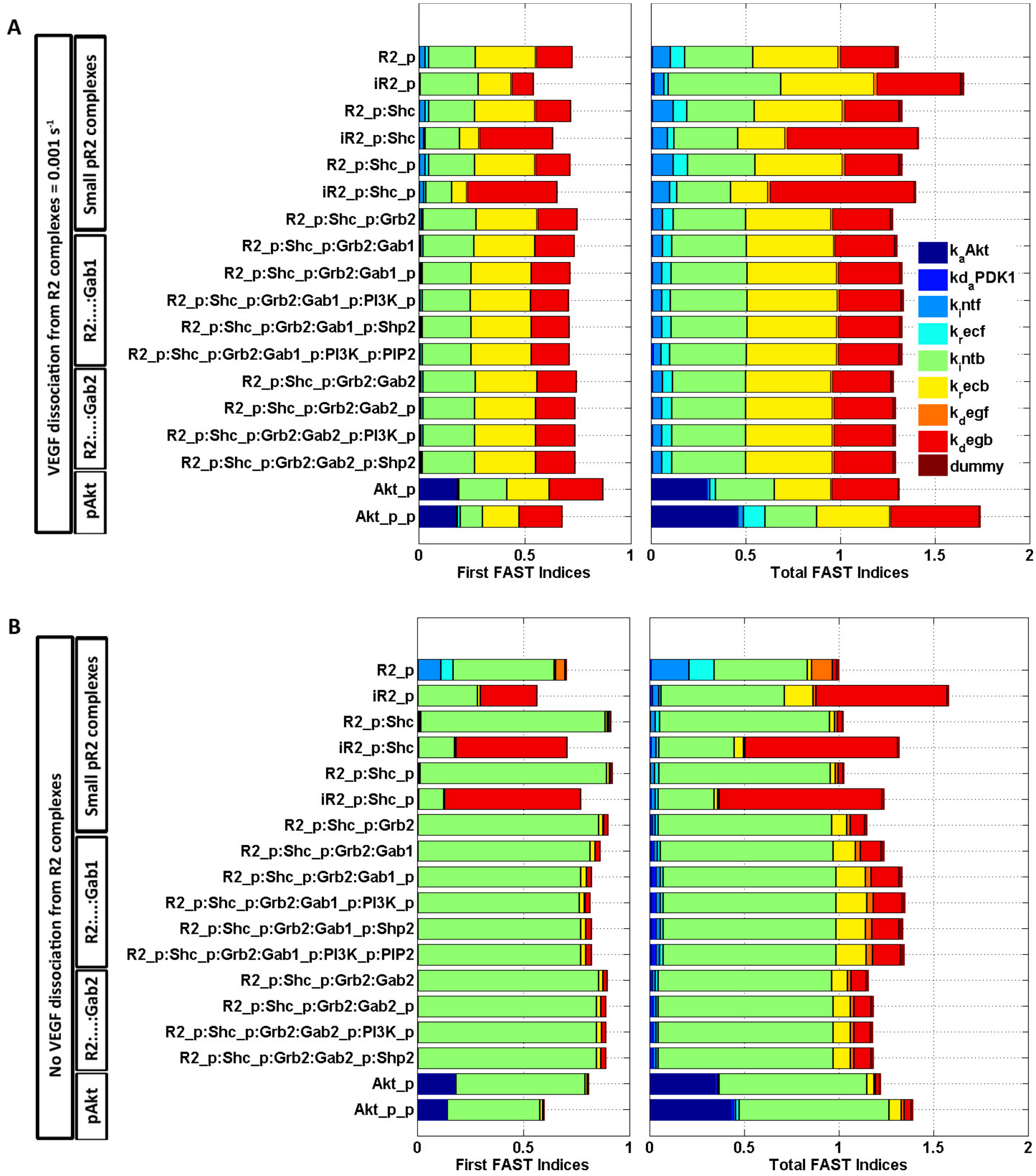

Figure S4: eFAST analysis of VEGFR2 trafficking parameters and Akt-phosphorylation parameters show that trafficking parameters are more sensitive. Dissociation of VEGF from VEGFR2-complexes increases the sensitivity of VEGFR2 recycling rates. (A) Current model (B) Previous version of the model where VEGF does not dissociate from VEGFR2-complexes.

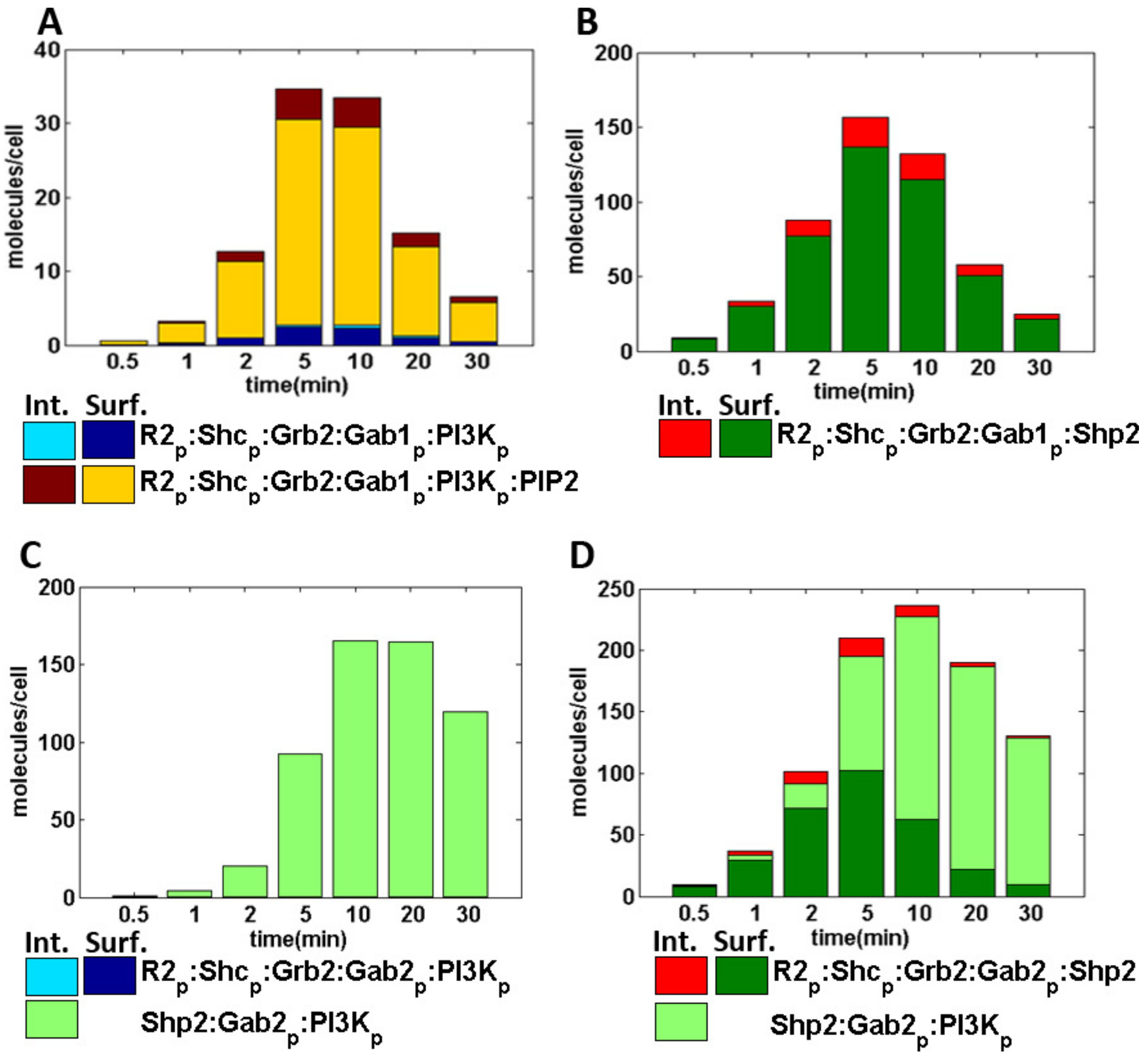

Figure S5: Shp2 dominates recruitment by Gab proteins. Model simulations for immunoprecipitates for Gab proteins followed by immunoblots for PI3K or Shp2. (A) Total Gab1 recruitment of PI3K. (B) Total Gab1 recruitment of Shp2. (C) Total Gab2 recruitment of PI3K. (D) Total Gab2 recruitment of Shp2. 'Int.' refers to internal, includes the receptor complexes in the endosomal pool and cytosol. 'Surf.' refers to plasma-membrane-associated receptor complexes on the cell surface.

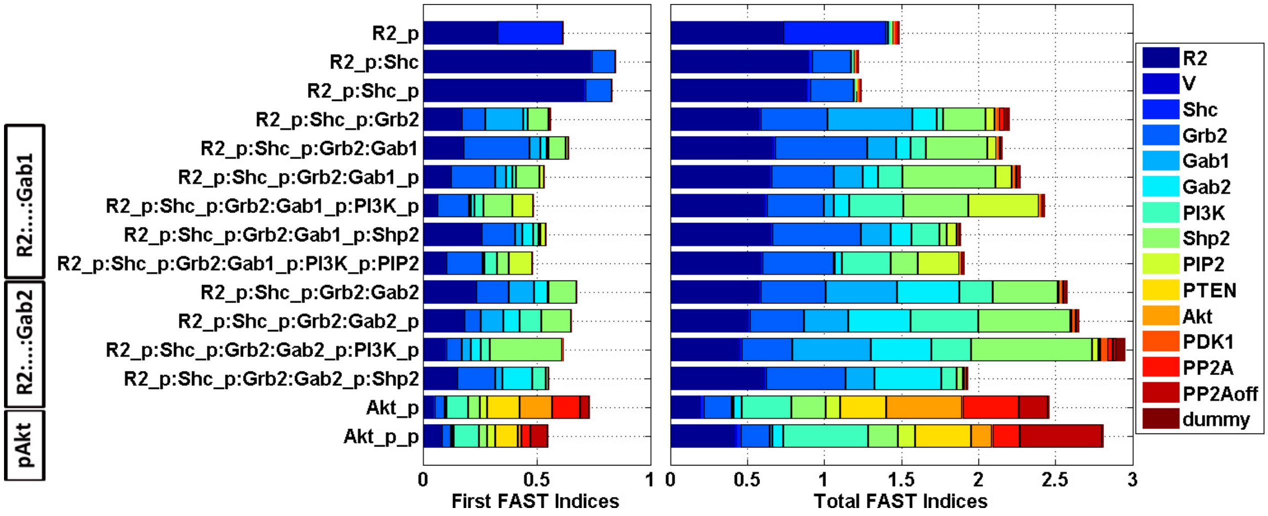

Figure S6. eFAST sensitivity indices of initial-concentration parameters. Internalized counterparts of receptor complexes not presented here have identical sensitivity indices as their membrane-associated counterparts.

Supplemental Methods

Sensitivity Analysis

With a large number of parameters in the model, it is important to use appropriate sensitivity analyses to determine which parameters play the largest role in determining the behavior of the system. Adjusting one parameter at a time ignores the interacting effects between parameters, but a joint adjustment of all parameters in all combinations is an exhaustive and costly search. Therefore, to better understand the interactions between parameters in the proposed mechanism, a more efficient algorithm is used; in this case, we perform variance-based global sensitivity analyses using the Extended Fourier Amplitude Sensitivity Test (eFAST) [42,43]. For this method, the model is run multiple times with different parameter sets each time. All parameters are varied from their baseline values for each run, but each parameter is varied at a different frequency j. Variance for a parameter i is calculated as:

$$D_i = 2\sum_{p=1}^{\infty}\left(A_{pj}^2 + B_{pj}^2\right)$$

where A_j and B_j are the Fourier coefficients of the cosine series and sine series respectively for the frequency (j) associated with the parameter i, and harmonics (p) of the base frequency are included;

$$A_j = \frac{1}{\pi}\int_{-\pi}^{\pi} f(x)\cos(jx)\,dx \qquad \text{and} \qquad B_j = \frac{1}{\pi}\int_{-\pi}^{\pi} f(x)\sin(jx)\,dx$$

where x is the input to the model and $f(x)$ is the output variable (calculated by the model) that is being evaluated for its sensitivity to variation in the parameters of the model.

The total variance in the output across all parameters is:

$$D_{total} = 2\sum_{j=1}^{\infty}\left(A_j^2 + B_j^2\right)$$

First-order FAST indices (labeled 'First FAST indices' in our figures) are measures of the local sensitivity, i.e. the sensitivity to changes in that parameter alone, ignoring second order interactions with other parameters:

$$S_i = \frac{D_i}{D_{total}}$$

Total FAST indices are measures of global sensitivity, taking into account second and higher order interactions between parameters of interest. They are calculated by excluding the effects of the complementary set of other parameters:

$$S_{Ti} = 1 - \frac{D_{ci}}{D_{total}}$$

For each output, the interaction between various inputs can be estimated by taking the difference between the total and the first indices for a particular input. Since kinetic parameters for the proposed mechanism have no precedent estimates, input parameters were sampled over a log-uniform distribution one order of magnitude greater and less than the baseline value, to cover a large, physiologically viable parameter space. 3000-4000 combinations of the input parameters were generated for this analysis, depending on the number of input parameters in each analysis. The minimum number of samples increases linearly with the number of input parameters, in order to avoid aliasing [44].
